# The causal relationship between sleep traits and the risk of schizophrenia: a two-sample bidirectional Mendelian randomization study

**DOI:** 10.1186/s12888-022-03946-8

**Published:** 2022-06-15

**Authors:** Zhen Wang, Miao Chen, Yin-ze Wei, Chen-gui Zhuo, Hong-fei Xu, Wei-dong Li, Liang Ma

**Affiliations:** 1 Department of Cardiovascular Surgery, School of Medicine, the First Affiliated Hospital of Zhejiang University, Number 79 Qingchun Road, Hangzhou, China; 2 Department of Cardiology, Taizhou Central Hospital (Taizhou University Hospital), Taizhou, Zhejiang China

**Keywords:** Sleep traits, Schizophrenia, Mendelian randomized study

## Abstract

**Background:**

Observational studies suggest that sleep disturbances are commonly associated with schizophrenia. However, it is uncertain whether this relationship is causal. To investigate the bidirectional causal relation between sleep traits and schizophrenia, we performed a two-sample bidirectional Mendelian randomization (MR) study with the fixed effects inverse-variance weighted (IVW) method.

**Methods:**

As genetic variants for sleep traits, we selected variants from each meta-analysis of genome-wide association studies (GWASs) conducted using data from the UK Biobank (UKB).

**Results:**

We found that morning diurnal preference was associated with a lower risk of schizophrenia, while long sleep duration and daytime napping were associated with a higher risk of schizophrenia. Multivariable MR analysis also showed that sleep duration was associated with a higher risk of schizophrenia after adjusting for other sleep traits. Furthermore, genetically predicted schizophrenia was negatively associated with morning diurnal preference and short sleep duration and was positively associated with daytime napping and long sleep duration.

**Conclusions:**

Therefore, sleep traits were identified as a potential treatment target for patients with schizophrenia.

**Supplementary Information:**

The online version contains supplementary material available at 10.1186/s12888-022-03946-8.

## Background

Schizophrenia is a chronic mental disorder with numerous combinations of nontypical symptoms, such as hallucinations, delusion and cognitive impairment [1]. According to statistical data, the prevalence rate of schizophrenia is approximately 0.28%; however, the prevalence rate depends on geographical variations [2, 3]. The recovery outcomes of schizophrenia are poor, and compared with that of healthy controls, schizophrenia significantly reduces an individual’s life expectancy by approximately 10 years on average [2].

Sleep dysfunction is prevalent in schizophrenia, is associated with distress and poorer clinical status, by report, as many as 80% of schizophrenia patients have experienced widespread sleep abnormalities (severe insomnia, increased sleep latency, reduced sleep continuity, sleep circadian rhythm disorder, and highly irregular and fragmented sleep patterns) [4, 5], however, it yet remains an under-recognized therapeutic target in clinical practice. According to the meta-analysis, there is a strong positive association between sleep dysfunction and schizophrenia [6]; though most meta-analyses have suggested potential causal associations between sleep dysfunction and schizophrenia, but it is difficult to confirm the causal relationship between sleep traits and schizophrenia.

Mendelian randomization (MR) is a method that uses genetic variants as instrumental variables to investigate the causal association between exposures and outcomes. MR may be considered conceptually as natural RCTs because genetic variants are randomly inherited. MR became an alternative approach to explore the potential causal relationship between an exposure and an outcome when randomized control trials are not feasible. Therefore, to investigate the bidirectional causal relation between sleep traits and schizophrenia in this study, we performed a two-sample bidirectional Mendelian randomization study to fill this gap and investigate the effect of sleep traits on patients with schizophrenia.

## Methods

### Study design

We conducted a two-sample bidirectional Mendelian randomization analysis to evaluate the bidirectional causal relationships between sleep traits and schizophrenia. The sleep traits included morning diurnal preference, sleep duration (short and long sleep duration), daytime sleepiness, daytime napping, and insomnia. Genetic summary statistics for exposures and outcomes were obtained from the largest available GWAS. All contributing GWASs sought informed consent from their study participants. Three assumptions must be satisfied in this MR analysis: 1) the genetic variant used in MR is associated with exposures; 2) associations of the genetic variant with sleep traits and schizophrenia must not be confounded; and 3) the genetic variants must have an association with outcomes only through the effect associated with exposures.

### Data sources and instruments selection

As genetic variants for morning diurnal preference, we selected variants from a meta-analysis of GWAS conducted using data from the UKB and 23andMe cohorts [7]. We only used the summary statistics from the UKB, which included 449,734 European individuals. The meta-analysis of GWASs identified 340 (*P* < 5 × 10^–8^) genetic loci, which were present in both UKB and 23andMe and were associated with morning diurnal preference. They also conducted a sensitivity analysis that excluded shift workers and those either on medication or with disorders affecting sleep. The summary statistics of continuous sleep duration were obtained from a recent GWAS in the UKB [8]. Through analysis in 446,118 European ancestry participants, the GWAS identified 78 genetic loci for self-reported habitual sleep duration, 27 genetic loci for short sleep duration (< 7 h), and long sleep durations (≥ 9 h) (*P* < 5 × 10^–8^). Sensitivity analyses indicated that these genetic associations were largely independent of known risk factors such as insomnia, caffeine, chronotype, additional lifestyle and clinical condition. Effect estimates were largely consistent in GWAS excluding shift workers and those with prevalent chronic and psychiatric disorders (excluding *n* = 119,894 participants). The summary statistics of daytime napping were obtained from a GWAS of self-reported daytime napping in the UKB (*n* = 452,633), and 123 distinct genetic loci were identified (*P* < 5 × 10^–8^) [9]. Effect estimates for these SNPs were largely consistent in GWAS restricted to 338,764 participants self-reporting excellent or good overall health. For daytime sleepiness, the summary-level data were extracted from a larger GWAS of self-reported daytime sleepiness [10]. They identified 42 loci for daytime sleepiness in GWAS of 452,071 individuals from the UKB. Sensitivity analyses were performed to adjust for potential confounders (including depression, socioeconomic status, alcohol intake frequency, smoking status, caffeine intake, employment status, marital status, neurodegenerative disorders, and psychiatric problems). They also used another GWAS (*N* = 255,426) that excluded shiftworkers and individuals with chronic health or psychiatric illnesses. The summary statistics of insomnia were extracted from a recent GWAS in the UKB including 453,379 European ancestry participants [11]. A total of 57 loci for self-reported insomnia symptoms were identified. The results from secondary GWAS excluding current shift workers or individuals reporting the use of hypnotic, antianxiolytic, or psychiatric medications and/or having selected chronic diseases or psychiatric illnesses (excluding *n* = 76,470 participants) were consistent with the primary GWAS. As genetic variants for schizophrenia, we extracted genetic variants from a large meta-analysis of GWAS including 77,096 European ancestry participants (33,640 cases and 43,456 controls) [12]. They identified 108 schizophrenia-associated genetic loci in their analysis (*P* < 5 × 10^–8^). The studies and datasets included in our analysis are presented in Supplementary Table 1. There was no participant overlap between the exposure dataset and outcomes datasets.

The linkage disequilibrium analysis among exposure-associated SNPs (single nucleotide polymorphism) was investigated using the clump function (r^2^ < 0.01 and clump window was 10,000 kb) in the TwoSampleMR package based on the 1000 Genomes LD (linkage disequilibrium) reference panel of only Europeans [13, 14]. We removed SNPs with effect sizes greater in the outcome than in the exposure (*P* < 5 × 10^–8^ in outcome). Among these, we selected 316 independent SNPs as instrumental variables for morning diurnal preference, 74 independent SNPs for sleep duration, 26 independent SNPs for short sleep duration, 7 independent SNPs for long sleep duration, 105 independent SNPs for daytime napping, 40 independent SNPs for daytime sleepiness, and 52 independent SNPs for insomnia (Supplementary Tables 2, 3, 4, 5, 6, 7 and 8). When schizophrenia was considered exposure, we selected 105 independent SNPs as instrumental variables (Supplementary Table 9).

If no matching SNP was available for an outcome, we selected proxies (r^2^ > 0.60) in LD Link (https://analysistools.cancer.gov/LDlink/?tab=home). Detailed information on the proxies used for genetic variables is presented in Supplementary Table 10. We excluded 6 SNPs (rs12249410, rs147439581, rs113397282, rs12055602, rs186545906, and rs114012503) because no proxy SNP was available. Finally, there were 315 SNPs as instrumental variables for morning diurnal preference, 101 SNPs as instrumental variables for schizophrenia, and 100 SNPs as instrumental variables for schizophrenia when the outcome was daytime napping.

### Statistical analysis

The causal relationship between sleep traits and schizophrenia was obtained from the fixed effects inverse-variance weighted (IVW) method. The Cochran’s Q statistic and the I^2^ statistic were performed to assess heterogeneity among estimates across individual SNPs. We considered there to be heterogeneity if *P* < 0.05 and used I^2^ to quantify heterogeneity (I^2^ ≤ 25%: small heterogeneity; 25% < I^2^ ≤ 50%: moderate heterogeneity; I^2^ ≥ 50%: larger heterogeneity). Leave-one-out analyses were conducted as a sensitivity analysis to preclude the possibility that the causal inference was driven by a single SNP.

Given that there was a positive correlation between sleep traits [10] and some genetic predictors predicting more than one sleep trait, we performed multivariable MR to explore the direct effects of morning diurnal preference, sleep duration, daytime napping, daytime sleepiness, and insomnia on schizophrenia by using the TwoSampleMR R package. We adjusted the thresholds for significance by Bonferroni correction. For the primary analyses (association of sleep traits with schizophrenia), we set 2-sided *P* values of < 0.007 (= 0.05/7 outcomes or exposures) as the thresholds for significance. All statistical analyses were conducted using the MRPRESSO and TwoSampleMR packages in R version 4.1.0 (R Core Team, Vienna, Austria).

## Results

### Causal effects of sleep traits on schizophrenia

We calculated the F-statistic to assess the instrument strength, and an F statistic greater than 10 was considered a strong genetic variant. The mean F statistic was 34 for morning diurnal preference, 40 for sleep duration, 25 for short sleep duration, 30 for long sleep duration, 46 for daytime napping, 41 for daytime sleepiness, and 39 for insomnia. Morning diurnal preference instruments explained an estimated 3.9% of phenotypic variability, sleep duration instruments explained an estimated 0.8% of phenotypic variability, short sleep duration instruments explained an estimated 1.2% of phenotypic variability, long sleep duration instruments explained an estimated 0.9% of phenotypic variability, daytime napping instruments explained an estimated 0.4% of phenotypic variability, daytime sleepiness instruments explained an estimated 0.1% of phenotypic variability, and insomnia instruments explained an estimated 0.2% of phenotypic variability. Given a type 1 error of 5%, there was > 80% power to detect significant differences at an odds ratio (OR) of 0.90 or lower for morning diurnal preference, an OR of 1.25 or higher for sleep duration, an OR of 1.20 or higher for short sleep duration, an OR of 1.24 or higher for long sleep duration, an OR of 1.37 or higher for daytime napping, an OR of 1.84 or higher for daytime sleepiness, and an OR of 1.55 or higher for insomnia.

**Fig. 1:**
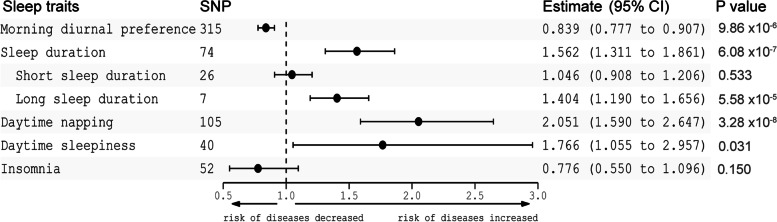
Mendelian randomization estimates for the association of sleep traits on schizophrenia. CI, confidence interval

In our primary analysis, genetically predicted morning diurnal preference was associated with a lower risk of schizophrenia (OR 0.839 [95% CI 0.777–0.907], *P* = 9.86 × 10^–6^) (Fig. 1). In addition, genetically predicted sleep duration (OR 1.562 [95% CI 1.311–1.861], *P* = 6.08 × 10^–7^) and daytime napping (OR 2.051 [95% CI 1.590–2.647], *P* = 3.28 × 10^–8^) were associated with a higher risk of schizophrenia (Fig. 1). However, our results suggested that daytime sleepiness (OR 1.766 [95% CI 1.055–2.957], *P* = 0.031) and insomnia (OR 0.776 [95% CI 0.550–1.096], *P* = 0.150) were not associated with a higher risk of schizophrenia (Fig. 1). Among sleep duration, our results showed that long sleep duration (OR 1.404 [95% CI 1.190–1.656], *P* = 5.58 × 10^–5^), but not short sleep duration (OR 1.046 [95% CI 0.908–1.206], *P* = 0.533), was associated with a higher risk of schizophrenia (Fig. 1, Supplementary Fig. 1).

The multiplicative random effects IVW, weighted median, and simple median methods were also conducted as sensitivity analyses to estimate the robustness of our results. The sensitivity analyses revealed that the associations were consistent with the primary results except for the association between daytime sleepiness and schizophrenia (Supplementary Table 11). We found that there was a larger heterogeneity for sleep traits with schizophrenia (Supplementary Table 12). The MR–Egger intercept analysis that was performed to detect directional pleiotropy did not indicate directional pleiotropy (Supplementary Table 12). MR-pleiotropy residual sum and outlier (MR-PRESSO) was adopted to detect outlier SNPs and to assess differences in the estimates before and after correction for outliers. The MR-PRESSO test showed outlier pleiotropy and suggested several SNP outliers in each sleep trait, but the results were consistent after correction for the SNP outliers (Supplementary Table 13). In the leave-one-out sensitivity analyses, the findings remained similar except for the estimate for the effect of daytime sleepiness, insomnia and schizophrenia (Supplementary Fig. 2A-G). The results showed that 8 SNPs influenced the IVW estimate for daytime sleepiness and schizophrenia, and 2 SNPs influenced the IVW estimate for insomnia and schizophrenia (Supplementary Fig. 2F, G).

**Fig. 2:**
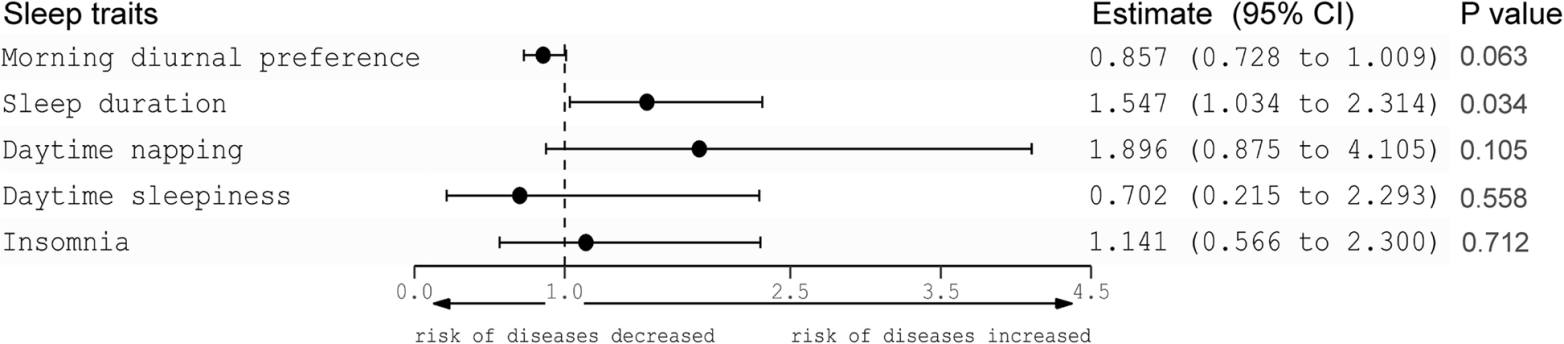
Multivariable MR estimates for morning diurnal preference, sleep duration, daytime napping, daytime sleepiness and insomnia on schizophrenia. CI, confidence interval

The results of multivariable MR showed that sleep duration was still associated with a higher risk of schizophrenia after adjusting for other sleep traits, morning diurnal preference and daytime napping were not clearly associated with schizophrenia, and the associations of daytime sleepiness and insomnia with schizophrenia were null (Fig. 2).

### Causal effects of schizophrenia on sleep traits

The mean F statistic was 41 for schizophrenia. The 105 schizophrenia instruments explained an estimated 27.1% of phenotypic variability. For schizophrenia, there was > 80% power to detect significant differences at an OR of 0.98 or lower for morning diurnal preference, at an OR of 0.98 or lower for short sleep duration, at an OR of 1.03 or higher for long sleep duration, at an OR of 1.016 or higher for daytime napping, at an OR of 1.019 or higher for daytime sleepiness, and at an OR of 1.018 or higher for insomnia.

**Fig. 3:**
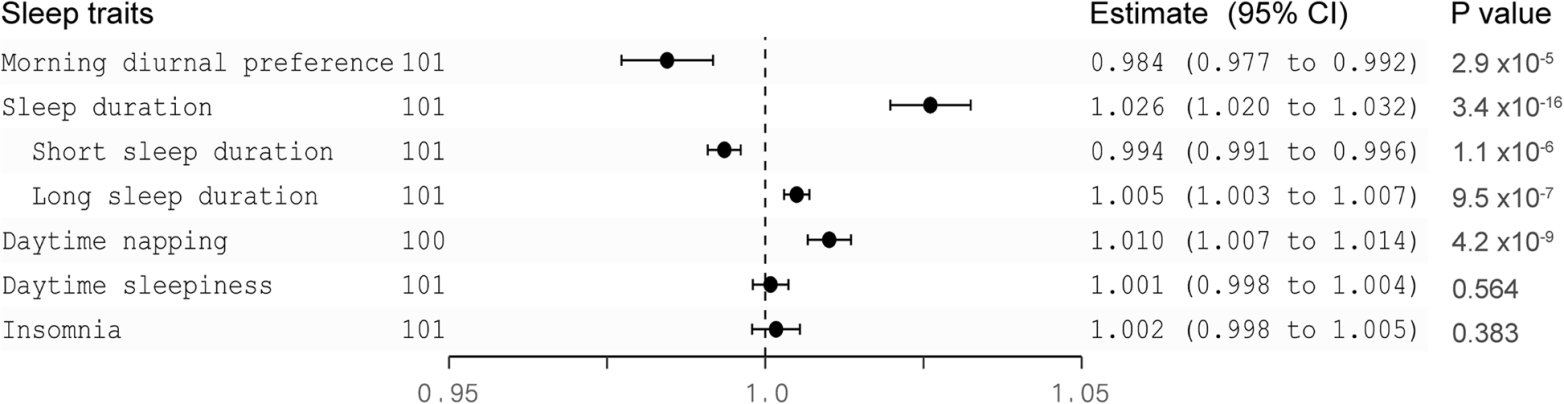
Mendelian randomization estimates for the association of schizophrenia on sleep traits. CI, confidence interval

Genetically predicted schizophrenia was negatively associated with morning diurnal preference (OR 0.984 [95% CI 0.977–0.992], *P* = 2.9 × 10^–5^), was associated with increased sleep duration (OR 1.026 [95% CI 1.020–1.032, *P* = 3.4 × 10^–16^) and was associated with higher odds of daytime napping (Fig. 3, Supplementary Fig. 3). The results showed that schizophrenia was not associated with daytime sleepiness (OR 1.001 [95% CI 0.998–1.004, *P* = 0.564) or insomnia (OR 1.002 [95% CI 0.998–1.005], *P* = 0.383). In sensitivity analyses, the associations were consistent by using the multiplicative random effects IVW, simple median, and weighted median methods (Supplementary Table 14). We also found that there was larger heterogeneity among sleep traits in schizophrenia (Supplementary Table 15). The MR–Egger intercept analysis did not indicate directional pleiotropy (Supplementary Table 15). The results of the MR-PRESSO test suggested several SNP outliers in schizophrenia, but the results were robust after correction for the SNP outliers (Supplementary Table 16). Finally, leave-one-out analyses showed no difference in our findings, illustrating that no single SNP influenced the estimates (Supplementary Fig. 4).

## Discussion

Our MR study revealed that morning diurnal preference was associated with a lower risk of schizophrenia; in addition, our results supplied clues for the causal relationship between long sleep duration and a higher risk of schizophrenia, between daytime napping and a higher risk of schizophrenia, and between daytime sleepiness and a higher risk of schizophrenia. On the other hand, consistent with previous studies, insomnia was not clearly causally associated with schizophrenia according to our Mendelian randomization results. Genetically predicted schizophrenia was negatively associated with morning diurnal preference, increased sleep duration and daytime napping.

The causal results of many modifiable exposures on disease outcomes that come from traditional observational epidemiological studies have proven to be unreliable. This is because traditional observational epidemiological studies are confounding, reverse causation and various biases. However, Mendelian randomization studies utilize genetic variants as instrumental variables that are robustly related to some modifiable exposures to reliably investigate whether the modifiable exposure factor causally affects health outcomes [15]. In contrast to observational studies, MR analysis especially MVMR analysis reduces the influence of confounders that may lead to spurious associations between exposures and outcomes. Our MR analyses support evidence for a causal link between sleep traits and schizophrenia, which is a supplement to the existing researches.

During the past decades, sleep abnormalities have been recognized as common features in schizophrenia, such as sleep disturbance, insomnia, nightmare, sleep circadian rhythm disorder, and highly irregular and fragmented sleep patterns [5, 16, 17]. Therefore, the potential importance of disrupted sleep in schizophrenia has attracted increasing recognition. Furthermore, there has been increasing interest in the distinct opinion that sleep abnormalities play a possible causal role in the outbreak and persistence of schizophrenia. However, because traditional observational studies are affected by confounding factors and biases, these studies could not clarify whether sleep abnormalities causally influence the risk of schizophrenia. Based on this setting, the MR study can confirm the causality of sleep traits in schizophrenia because of its characteristics.

Consistent with previous studies, our MR results revealed that insomnia showed no definite causality for schizophrenia. In addition, our results determined that morning diurnal preference was associated with a lower risk of schizophrenia, and our results supplied clues for the causal relationship between long sleep duration, daytime napping, daytime sleepiness and a higher risk of schizophrenia. Our findings strongly support the view that sleep traits can be a treatment target in schizophrenia patients.

The significant advantage of our study is the large sample size of the resulting GWAS meta-analyses, which provides considerable statistical accuracy. In addition, the estimation of MR was strong based on the sensitivity analysis in a variety of methods that tested for stability and model violations. One limitation of our study was that SNPs in the exposure variable examined did not adequately account for phenotypic variance. However, the statistical power in our study was adequate to identify the effect sizes for sleep traits and schizophrenia outcomes, and all F statistics suggested the chosen tools as credible instruments, where 10 indicates weak instrument bias. In the future, when a larger GWAS is available, it will contribute to supplying more SNPs that could be used as exposure variables and enhancing the accuracy of our MR estimates.

## Limitations

At first, the most significant of our limitations is the limited representation of non-European ancestry samples in UK Biobank repositories. Second, a few more risk loci for schizophrenia have been identified since 2014, but we did not include these in our manuscript. Finally, we did not exclude those with schizophrenia on psychiatric medications, because the effect estimates were largely consistent in GWAS excluding shift workers and those with prevalent chronic and psychiatric disorders (excluding *n* = 119,894 participants).

## Supplementary Information

**Additional file 1.**

**Additional file 2.**

**Additional file 3.**

**Additional file 4.**

**Additional file 5.**

## Data Availability

All data generated and/or analyzed during this study are included in this published article.
