# Supplementary figures and images for: The causal relationship between sleep traits and the risk of schizophrenia: a two-sample bidirectional Mendelian randomization study
Source: BMC Psychiatry. 2022 Jun 15;22:399. doi: 10.1186/s12888-022-03946-8 (PMC9202113; doi:10.1186/s12888-022-03946-8)

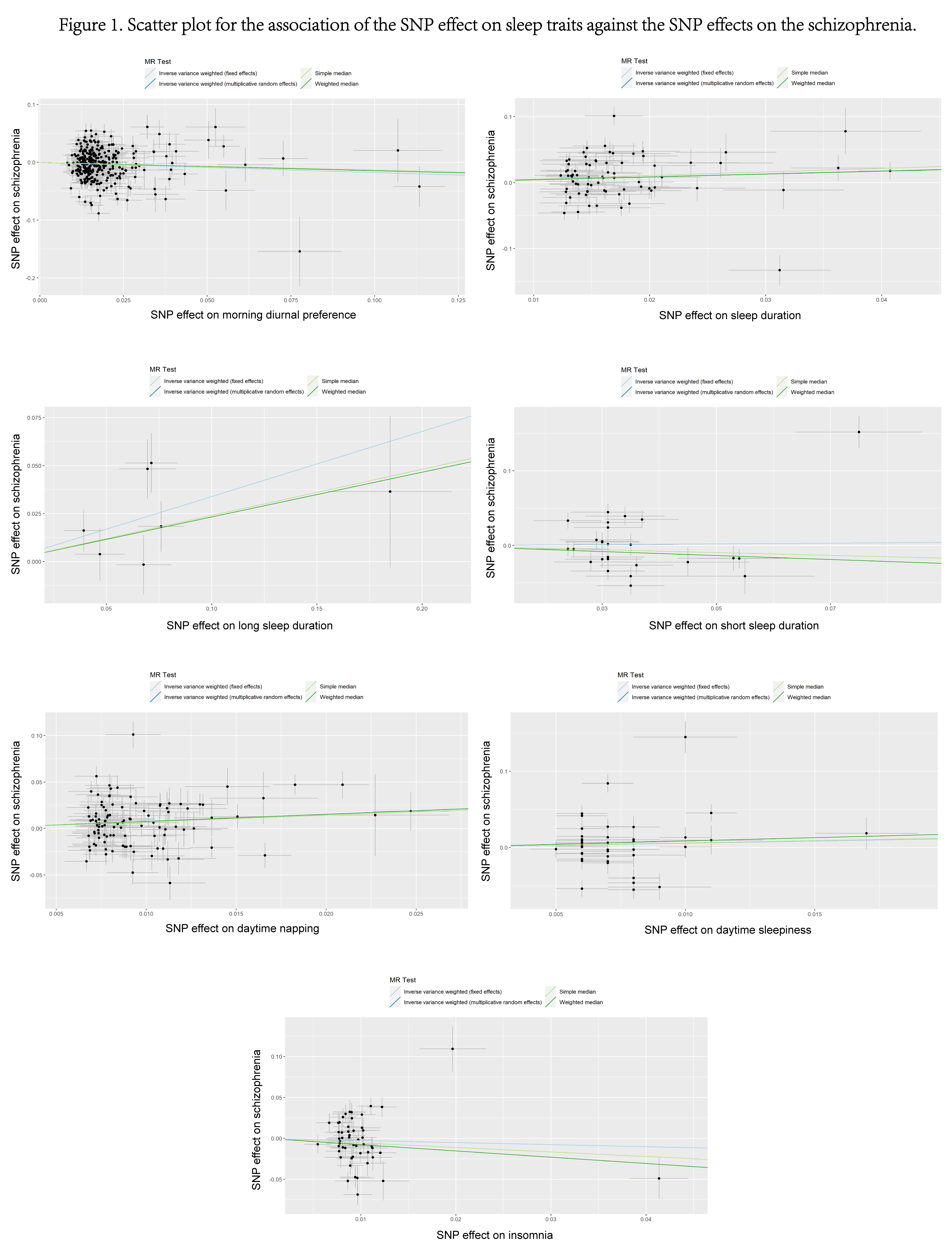

Supplement: Supplementary file 1 — Additional file 1. [file 12888_2022_3946_MOESM1_ESM.tif]

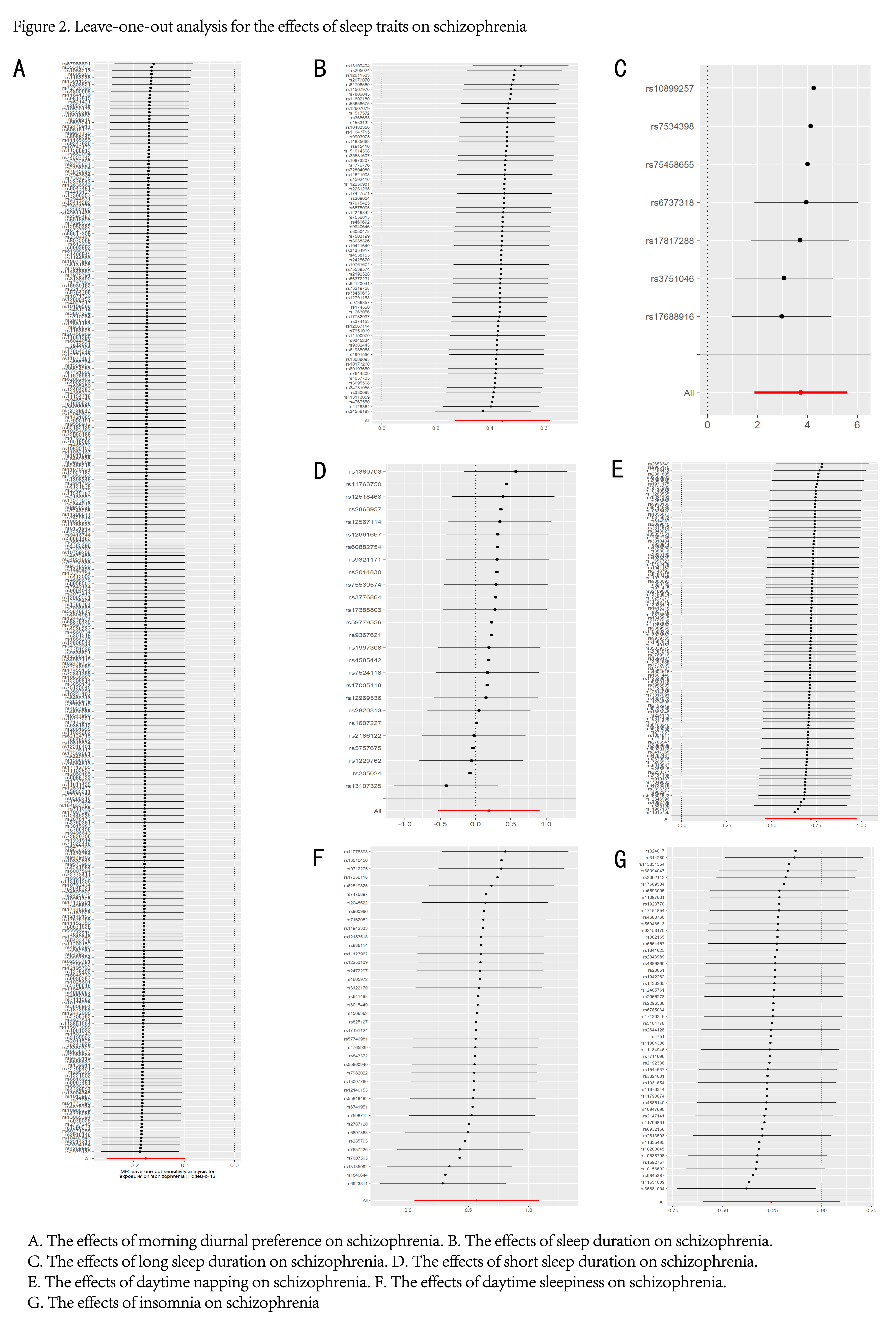

Supplement: Supplementary file 2 — Additional file 2. [file 12888_2022_3946_MOESM2_ESM.tif]

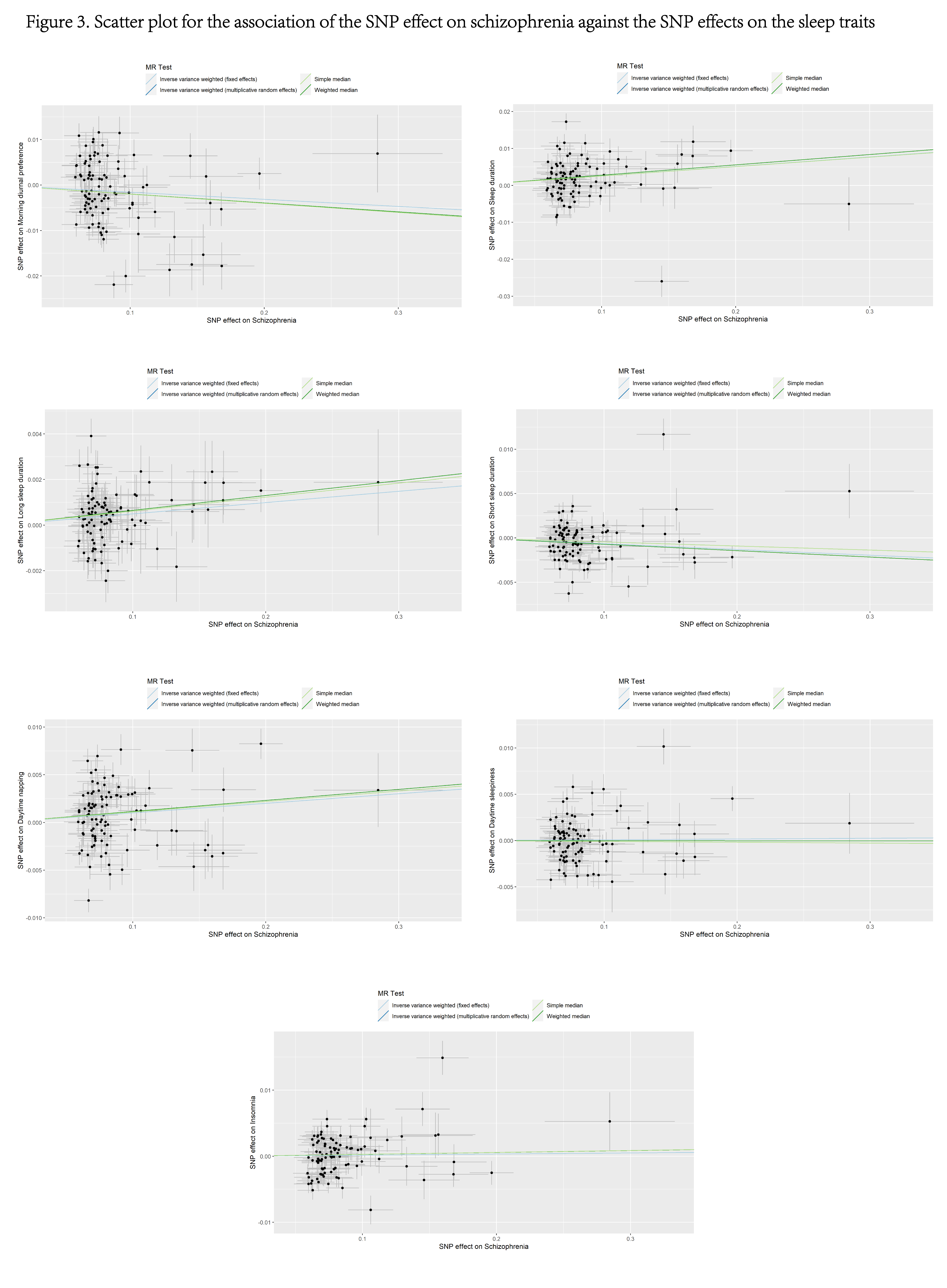

Supplement: Supplementary file 3 — Additional file 3. [file 12888_2022_3946_MOESM3_ESM.tif]

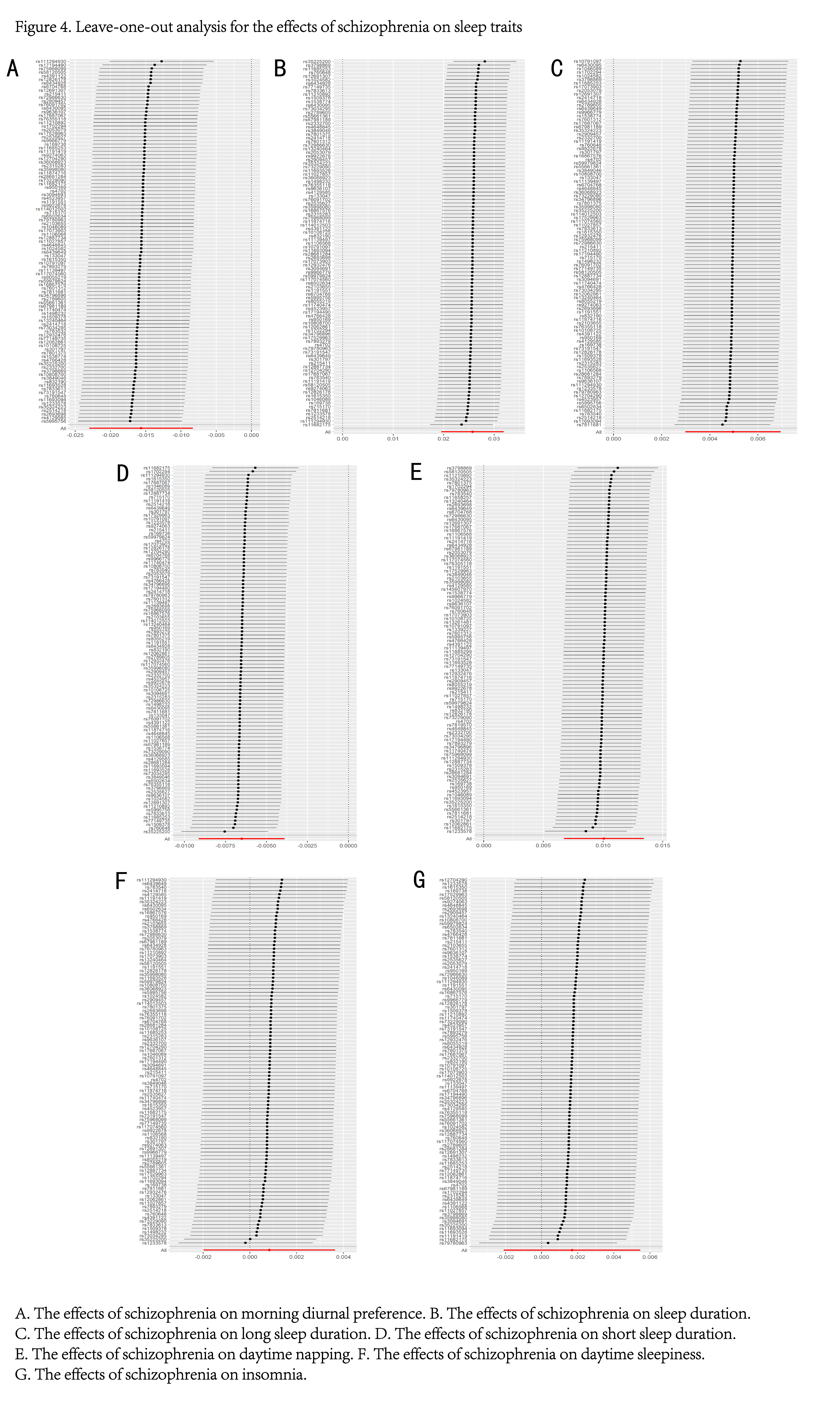

Supplement: Supplementary file 4 — Additional file 4. [file 12888_2022_3946_MOESM4_ESM.tif]
