# Supplementary material for: The causal relationship between sleep traits and the risk of schizophrenia: a two-sample bidirectional Mendelian randomization study
Source: BMC Psychiatry. 2022 Jun 15;22:399. doi: 10.1186/s12888-022-03946-8 (PMC9202113; doi:10.1186/s12888-022-03946-8)
Supplement: Supplementary file 5 — Additional file 5. [file 12888_2022_3946_MOESM5_ESM.pdf]

Table 1. Descriptive information of the studies and datasets included in the analyses.

GWAS	Phenotype	Participants	Ancestry
Jones SE, 2019*	Morning diurnal preference	449,734 individuals	European
Dashti HS, 2019	Sleep duration/ Long sleep duration/ Short sleep duration	446,118 individuals	European
Dashti HS, 2021	Daytime napping	452,633 individuals	European
Wang H, 2019	Daytime sleepiness	452,071 individuals	European
Lane J, 2019	Isomnia	453,379 individuals	European
Psychiatric Genomics Consortium	Schizophrenia	33,640 cases 43,456 controls	European

* We used the set of UK Biobank-only chronotype GWAS summary statistics.

Table 2. Associations of single nucleotide polymorphisms for morning diurnal preference. Chr, chromosome; EA, effect allele; NEA, non-effect allele; EAF, effect allele frequency.

SNP	Chr	Position	EA	NEA	EAF	Beta	SE	P-value*	F-statistic	R^2
rs61773390	1	7884525	G	T	0.80	-0.0341	0.0034	1.20E-23	101.9	0.0004
rs12065331	1	14507831	C	T	0.69	0.0159	0.0029	6.70E-08	29.5	0.0001
rs17448682	1	15966713	C	T	0.77	-0.0222	0.0032	2.20E-12	48.5	0.0002
rs10916892	1	21201325	T	C	0.63	-0.0200	0.0028	6.10E-13	51.7	0.0002
rs12140153	1	62579891	G	T	0.90	0.0340	0.0047	1.60E-13	52.6	0.0002
rs11208844	1	66851147	G	A	0.86	0.0191	0.0039	1.30E-06	24.1	0.0001
rs12040629	1	77705365	G	A	0.84	-0.0392	0.0036	6.70E-27	115.2	0.0004
rs11588913	1	79963816	G	A	0.60	0.0155	0.0027	1.50E-08	31.8	0.0001
rs5016898	1	81672013	C	T	0.57	0.0123	0.0027	5.80E-06	20.2	0.0001
rs72720396	1	91191582	A	G	0.77	-0.0271	0.0032	8.40E-18	72.5	0.0003
rs481214	1	93469865	A	T	0.60	0.0132	0.0027	8.70E-07	23.3	0.0001
rs11165655	1	96959104	G	A	0.48	0.0157	0.0027	4.10E-09	34.2	0.0001
rs17575798	1	110086451	G	A	0.81	0.0226	0.0034	4.20E-11	44.2	0.0002
rs6690292	1	113188419	C	T	0.27	0.0135	0.0030	4.50E-06	19.9	0.0001
rs11102807	1	115061584	A	G	0.54	-0.0131	0.0027	2.10E-07	23.4	0.0001
rs9436119	1	150467753	G	A	0.61	-0.0235	0.0027	4.00E-18	73.6	0.0003
rs6665637	1	153756083	G	A	0.72	0.0152	0.0030	6.20E-07	24.8	0.0001
rs115073088	1	174215858	A	G	0.98	-0.0525	0.0089	3.10E-09	34.5	0.0001
rs975025	1	179338327	C	T	0.92	0.0321	0.0050	1.30E-10	40.8	0.0001
rs1144566	1	182569626	T	C	0.03	0.1134	0.0078	5.80E-47	209.3	0.0008
rs1221502	1	193276975	A	C	0.74	0.0150	0.0031	8.30E-07	24.2	0.0001
rs4657983	1	195454557	G	A	0.35	0.0136	0.0028	1.50E-06	23.1	0.0001
rs6429233	1	241137033	G	A	0.55	-0.0129	0.0027	2.10E-06	22.9	0.0001
rs13011556	2	4651923	C	G	0.76	-0.0227	0.0032	1.10E-12	51.8	0.0002
rs62124718	2	12822995	A	G	0.89	-0.0228	0.0044	1.20E-07	27.2	0.0001

rs72796401	2	24180078	T	A	0.81	-0.0220	0.0034	5.90E-11	41	0.0001
rs11678584	2	32563426	A	T	0.86	-0.0170	0.0039	2.80E-05	19.2	0.0001
rs848552	2	36700580	C	G	0.47	-0.0153	0.0027	7.00E-09	32.5	0.0001
rs75120545	2	44271496	C	T	0.97	-0.0614	0.0083	1.10E-13	54.9	0.0002
rs6544906	2	46863872	A	C	0.56	0.0119	0.0027	1.10E-05	19.4	0.0001
rs17396357	2	48252311	C	T	0.62	-0.0093	0.0028	6.10E-04	11.3	4.1E-05
rs12470914	2	50532840	T	A	0.90	-0.0261	0.0044	2.20E-09	34.6	0.0001
rs4672458	2	53736362	C	T	0.53	0.0163	0.0027	1.10E-09	36.7	0.0001
rs13414393	2	54275162	T	C	0.54	-0.0134	0.0027	8.10E-07	24.6	0.0001
rs10175975	2	59429807	C	T	0.82	-0.0228	0.0035	1.80E-10	42.3	0.0002
rs359248	2	60477461	T	G	0.45	-0.0162	0.0027	1.50E-09	35.5	0.0001
rs812925	2	61680993	C	G	0.65	-0.0206	0.0028	6.90E-13	53.6	0.0002
rs113851554	2	66750564	G	T	0.94	0.0357	0.0060	1.00E-09	35.6	0.0001
rs2706762	2	70488470	C	T	0.85	0.0219	0.0038	4.00E-09	34.1	0.0001
rs12464387	2	75445544	A	G	0.46	-0.0137	0.0027	4.70E-07	25.7	0.0001
rs6727752	2	76361783	G	A	0.63	-0.0157	0.0028	1.40E-08	32	0.0001
rs10520176	2	77217310	T	C	0.50	0.0231	0.0027	1.40E-17	74.1	0.0003
rs11681299	2	88901732	C	T	0.71	-0.0111	0.0030	2.70E-04	14.1	0.0001
rs34509802	2	101591710	G	A	0.82	-0.0154	0.0035	5.30E-06	19.3	0.0001
rs76064513	2	125438641	C	T	0.87	-0.0154	0.0041	1.50E-04	14.4	0.0001
rs77248969	2	136490731	G	A	0.91	0.0218	0.0047	1.80E-06	21.5	0.0001
rs28380327	2	144232491	A	T	0.63	0.0205	0.0028	2.90E-13	54.5	0.0002
rs2166559	2	149551658	T	C	0.86	-0.0218	0.0039	9.10E-09	31.8	0.0001
rs747003	2	161916409	T	C	0.61	0.0146	0.0028	2.70E-07	28.1	0.0001
rs13004345	2	174037347	C	T	0.35	0.0132	0.0028	2.00E-06	22.1	0.0001
rs6433478	2	175241482	T	C	0.45	-0.0120	0.0027	5.30E-06	19.6	0.0001
rs4666682	2	186203743	G	A	0.82	0.0173	0.0035	1.00E-06	24.2	0.0001
rs11677484	2	191578172	G	T	0.74	-0.0186	0.0031	1.70E-09	36	0.0001
rs1064213	2	198950240	G	A	0.52	-0.0191	0.0027	1.30E-12	50.8	0.0002
rs184033703	2	206956138	G	A	0.94	-0.0378	0.0058	8.10E-11	43	0.0002
rs80271258	2	239311505	C	T	0.91	0.0549	0.0048	2.60E-30	132.8	0.0005
rs62182135	2	240267305	C	A	0.67	0.0179	0.0028	3.70E-10	39.7	0.0001
rs35346733	3	2521322	G	A	0.81	0.0176	0.0034	4.50E-07	26.5	0.0001
rs149611468	3	8817423	T	C	0.99	0.0776	0.0126	8.50E-10	38	0.0001
rs6794796	3	14383632	A	G	0.29	0.0172	0.0030	5.30E-09	33.7	0.0001
rs9817910	3	18246870	G	A	0.44	0.0170	0.0027	3.10E-10	39.6	0.0001
rs73050286	3	23224684	T	C	0.78	0.0178	0.0033	2.60E-08	29.8	0.0001
rs2362775	3	24924421	T	C	0.53	-0.0180	0.0027	2.80E-11	44.5	0.0002
rs114848860	3	36859494	A	T	0.98	-0.0556	0.0087	3.60E-10	40.6	0.0001
rs12636669	3	50003323	C	T	0.92	-0.0343	0.0050	5.80E-12	47.7	0.0002
rs17007397	3	70594975	C	G	0.58	0.0109	0.0027	7.20E-05	15.9	0.0001
rs7626335	3	71575177	A	C	0.33	-0.0115	0.0029	3.00E-05	16	0.0001

rs7429614	3	77205438	G	T	0.58	-0.0164	0.0027	9.20E-10	36.4	0.0001
rs12631477	3	83804561	T	C	0.80	0.0169	0.0034	7.90E-07	25.2	0.0001
rs1449403	3	85591467	G	A	0.88	-0.0197	0.0041	6.10E-07	23.4	0.0001
rs34967119	3	104778430	G	A	0.50	-0.0127	0.0027	3.80E-06	22.4	0.0001
rs1398346	3	110271943	C	T	0.13	-0.0175	0.0040	1.50E-05	19	0.0001
rs1800828	3	113891549	C	G	0.75	0.0176	0.0031	1.70E-08	32.6	0.0001
rs72950188	3	116103275	T	C	0.92	0.0241	0.0051	2.00E-06	22.4	0.0001
rs72966564	3	123149816	C	T	0.75	0.0174	0.0031	2.50E-08	31.1	0.0001
rs13065394	3	132971327	G	T	0.71	0.0175	0.0030	3.00E-09	34.9	0.0001
rs7649164	3	150788032	T	G	0.58	0.0134	0.0028	2.30E-06	23.4	0.0001
rs6440833	3	152646244	G	A	0.54	-0.0120	0.0027	1.00E-05	19.8	0.0001
rs35588117	3	157817438	A	G	0.11	-0.0212	0.0044	9.30E-07	23.2	0.0001
rs1599374	3	160891727	G	A	0.49	-0.0154	0.0027	1.50E-08	32.4	0.0001
rs3850174	3	172364093	T	A	0.74	0.0194	0.0031	6.80E-10	39.4	0.0001
rs301218	3	176096919	G	A	0.61	0.0122	0.0027	1.00E-05	19.7	0.0001
rs9836621	3	182096311	C	T	0.48	0.0127	0.0027	4.80E-06	22.2	0.0001
rs1468945	3	185990392	G	A	0.21	0.0226	0.0033	4.20E-12	47.7	0.0002
rs3796618	4	1349602	T	A	0.47	0.0133	0.0027	5.50E-07	24.2	0.0001
rs4690085	4	2697300	A	G	0.53	-0.0147	0.0027	3.90E-08	30	0.0001
rs4698678	4	18260776	C	G	0.28	0.0140	0.0030	2.70E-06	22.1	0.0001
rs1502249	4	27495379	A	G	0.52	0.0122	0.0027	4.80E-06	20.4	0.0001
rs6838677	4	66520667	A	C	0.67	-0.0134	0.0029	2.80E-06	21.9	0.0001
rs4860734	4	67096904	G	A	0.72	-0.0137	0.0030	6.80E-06	20.6	0.0001
rs6816922	4	80206272	C	A	0.46	0.0116	0.0027	1.20E-05	18.3	0.0001
rs6846730	4	83279041	C	T	0.77	0.0220	0.0032	5.60E-12	47.9	0.0002
rs2850979	4	102094764	C	T	0.24	0.0167	0.0032	7.90E-08	27.9	0.0001
rs7700110	4	114439894	G	A	0.74	-0.0134	0.0031	1.30E-05	19	0.0001
rs17455138	4	130903511	T	C	0.77	0.0130	0.0032	3.50E-05	16.7	0.0001
rs4241964	4	137053959	T	G	0.52	-0.0207	0.0027	1.10E-14	58.8	0.0002
rs938836	4	139939653	G	A	0.53	0.0130	0.0027	2.50E-06	23.4	0.0001
rs72729847	4	147296930	T	C	0.80	-0.0159	0.0034	1.70E-06	22	0.0001
rs9997394	4	163704083	G	A	0.71	0.0136	0.0030	8.30E-06	21.2	0.0001
rs10058356	5	35220404	C	T	0.30	0.0170	0.0029	6.80E-09	33.5	0.0001
rs7701529	5	63861475	A	T	0.24	-0.0188	0.0032	3.60E-09	35.3	0.0001
rs7721608	5	76581258	G	T	0.54	-0.0114	0.0027	4.20E-05	17.8	0.0001
rs4269995	5	87701223	C	T	0.75	0.0213	0.0031	5.90E-12	47.4	0.0002
rs77960	5	103964585	G	A	0.67	-0.0188	0.0029	4.20E-11	42.9	0.0002
rs1559253	5	106657015	G	A	0.64	-0.0097	0.0028	7.70E-04	11.8	4.3E-05
rs17140201	5	115939896	G	A	0.83	0.0194	0.0036	1.20E-07	28.6	0.0001
rs67988891	5	152204741	C	G	0.68	-0.0232	0.0029	3.70E-15	64.7	0.0002
rs2901796	5	163330708	A	G	0.40	0.0135	0.0028	7.00E-07	24.1	0.0001
rs42210	5	166408788	G	C	0.29	0.0137	0.0030	3.00E-06	21.1	0.0001

rs12518401	5	173539588	G	A	0.61	0.0125	0.0028	7.00E-06	19.4	0.0001
rs465670	5	176877624	C	T	0.46	-0.0145	0.0027	6.30E-08	28.9	0.0001
rs9394154	6	11574374	C	G	0.43	-0.0155	0.0027	1.30E-08	32.6	0.0001
rs9381812	6	13183998	A	G	0.70	-0.0231	0.0029	4.60E-15	61.2	0.0002
rs1811899	6	14878060	T	C	0.79	-0.0138	0.0033	4.00E-05	17.4	0.0001
rs9465253	6	19102247	C	T	0.72	-0.0144	0.0030	1.40E-06	23	0.0001
rs766406	6	26319588	G	T	0.37	0.0115	0.0028	7.10E-05	17	0.0001
rs605203	6	31847012	C	A	0.37	0.0152	0.0028	3.80E-08	29.8	0.0001
rs3923809	6	38440970	A	G	0.70	-0.0114	0.0029	1.10E-04	15.3	0.0001
rs12206814	6	41517457	G	C	0.51	-0.0152	0.0027	2.80E-08	30.8	0.0001
rs2396004	6	43355851	A	G	0.43	0.0125	0.0027	4.30E-06	21.2	0.0001
rs3857599	6	50938247	C	A	0.84	-0.0219	0.0036	1.40E-09	36.1	0.0001
rs2653349	6	55142337	A	G	0.21	0.0387	0.0033	5.20E-32	139.9	0.0005
rs1931814	6	62589167	A	G	0.48	0.0154	0.0027	1.60E-08	32.7	0.0001
rs2881955	6	72479263	C	T	0.72	-0.0161	0.0030	4.30E-08	28.8	0.0001
rs12195792	6	98705295	T	A	0.73	-0.0149	0.0030	6.70E-07	24.1	0.0001
rs11154718	6	99592404	T	C	0.43	-0.0121	0.0027	5.80E-06	19.9	0.0001
rs60616179	6	110244765	A	G	0.95	0.0376	0.0059	1.10E-10	39.9	0.0001
rs4535583	6	115699280	C	T	0.30	-0.0122	0.0029	3.90E-05	17.5	0.0001
rs9496643	6	143789040	G	A	0.71	-0.0134	0.0030	9.00E-06	20.3	0.0001
rs2050185	6	147936781	G	A	0.37	-0.0149	0.0028	8.90E-08	28.6	0.0001
rs9479402	6	153135339	T	C	0.99	-0.1069	0.0132	3.20E-16	65.7	0.0002
rs9347926	6	165195547	A	T	0.45	0.0157	0.0027	7.30E-09	33.9	0.0001
rs9348050	6	166263488	T	C	0.49	0.0166	0.0027	7.50E-10	38.2	0.0001
rs4027217	7	14093914	C	A	0.79	0.0150	0.0033	3.00E-06	21	0.0001
rs10237162	7	24085405	T	C	0.73	0.0176	0.0030	3.50E-09	34.2	0.0001
rs10951325	7	32265545	T	C	0.63	0.0206	0.0028	7.90E-14	54	0.0002
rs6967481	7	50642701	C	T	0.50	-0.0206	0.0027	6.90E-15	58.5	0.0002
rs4236237	7	69936477	C	A	0.40	0.0128	0.0027	2.80E-06	22	0.0001
rs2944831	7	71779635	G	A	0.70	-0.0172	0.0030	1.10E-08	34.1	0.0001
rs3807651	7	77823771	A	T	0.49	0.0129	0.0027	1.40E-06	22.9	0.0001
rs10254050	7	96468077	C	G	0.19	-0.0246	0.0034	1.20E-12	51.2	0.0002
rs4729854	7	102383663	T	A	0.52	0.0287	0.0028	3.30E-25	109	0.0004
rs6961970	7	113901132	C	A	0.75	-0.0165	0.0031	1.80E-07	27.8	0.0001
rs17302081	7	115673079	T	C	0.44	0.0157	0.0027	6.90E-09	33.5	0.0001
rs6968240	7	121942674	C	A	0.57	-0.0118	0.0027	1.80E-05	18.7	0.0001
rs62465218	7	132294312	C	A	0.85	0.0152	0.0038	4.20E-05	16.3	0.0001
rs6958557	7	133585794	T	G	0.61	0.0127	0.0028	1.70E-06	21.3	0.0001
rs113161209	7	148564367	G	A	0.93	-0.0246	0.0053	4.50E-06	21.4	0.0001
rs2072413	7	150647969	C	T	0.73	0.0142	0.0030	3.50E-06	21.8	0.0001
rs62479736	8	3654320	T	G	0.29	0.0152	0.0030	1.20E-07	26.5	0.0001
rs35524253	8	4823608	G	A	0.64	-0.0159	0.0028	5.80E-09	31.8	0.0001

rs2979139	8	8268313	A	G	0.51	-0.0168	0.0027	7.40E-10	38.9	0.0001
rs2322605	8	27164449	G	A	0.52	0.0126	0.0027	2.50E-06	21.9	0.0001
rs71523448	8	31817493	G	C	0.92	0.0280	0.0051	2.00E-08	30.7	0.0001
rs6993892	8	33729200	T	C	0.61	-0.0205	0.0028	8.40E-14	55.4	0.0002
rs6468316	8	35237788	C	T	0.52	0.0148	0.0027	5.90E-08	30.4	0.0001
rs7845620	8	53129069	A	C	0.84	-0.0225	0.0036	5.20E-10	38.3	0.0001
rs10109566	8	59800446	A	G	0.49	-0.0131	0.0027	1.50E-06	23.6	0.0001
rs34054660	8	65015659	A	G	0.57	0.0087	0.0027	2.20E-03	10.3	3.7E-05
rs187028	8	73459513	A	T	0.32	-0.0151	0.0029	3.10E-07	27.3	0.0001
rs16939162	8	76653156	A	G	0.83	0.0250	0.0036	2.20E-12	48.6	0.0002
rs6988733	8	91535686	C	T	0.65	-0.0121	0.0028	2.00E-05	18.5	0.0001
rs7006885	8	93283578	G	A	0.71	-0.0100	0.0030	9.40E-04	11.4	4.1E-05
rs3100052	8	101967139	A	G	0.39	0.0171	0.0028	6.90E-10	38.3	0.0001
rs2737245	8	116658583	G	T	0.72	-0.0215	0.0030	4.50E-13	51.3	0.0002
rs1871729	8	136223702	A	G	0.68	-0.0126	0.0029	7.20E-06	18.9	0.0001
rs6477309	9	8450638	C	T	0.33	-0.0174	0.0029	8.00E-10	37.1	0.0001
rs2844016	9	24582747	T	C	0.30	0.0132	0.0029	1.30E-05	20	0.0001
rs308521	9	37367094	T	C	0.60	0.0200	0.0028	3.70E-13	52.6	0.0002
rs4878734	9	38010085	A	T	0.52	0.0139	0.0027	3.70E-07	26.7	0.0001
rs6560218	9	74245426	C	T	0.48	0.0128	0.0027	1.30E-06	22.3	0.0001
rs62553781	9	76679777	C	T	0.97	0.0504	0.0073	1.20E-11	47.1	0.0002
rs12378543	9	83196097	C	T	0.61	0.0135	0.0028	6.60E-07	23.7	0.0001
rs555784	9	85318704	T	A	0.62	0.0130	0.0028	1.80E-06	22.2	0.0001
rs295268	9	86429305	T	C	0.74	-0.0166	0.0031	4.10E-08	29	0.0001
rs3138490	9	92219000	T	A	0.48	-0.0139	0.0027	1.90E-07	26.2	0.0001
rs10759208	9	109806199	T	C	0.61	-0.0132	0.0028	6.00E-07	22.9	0.0001
rs11788633	9	116767656	C	G	0.65	0.0117	0.0028	4.00E-05	17	0.0001
rs10818834	9	126317324	T	C	0.73	0.0189	0.0030	1.10E-09	38.3	0.0001
rs10988239	9	131943440	C	T	0.49	0.0186	0.0027	1.60E-11	46.5	0.0002
rs12380242	9	139310187	T	C	0.50	-0.0158	0.0027	1.60E-09	34.6	0.0001
rs28458909	9	140257189	C	T	0.88	0.0433	0.0041	2.60E-26	112.7	0.0004
rs497338	10	804315	C	T	0.72	-0.0149	0.0030	6.90E-07	25	0.0001
rs66617308	10	56699338	T	C	0.67	0.0144	0.0029	2.30E-07	25.4	0.0001
rs9416744	10	60567937	A	C	0.26	0.0190	0.0031	1.30E-09	38	0.0001
rs12249410	10	64301941	G	T	0.89	0.0237	0.0044	6.40E-08	29.3	0.0001
rs17712705	10	69623271	A	G	0.33	-0.0126	0.0029	1.40E-05	19.4	0.0001
rs2298117	10	70346740	T	C	0.45	-0.0151	0.0027	5.10E-08	31.2	0.0001
rs10762434	10	73044413	G	C	0.22	-0.0124	0.0032	1.10E-04	14.6	0.0001
rs2648721	10	93026996	T	G	0.70	-0.0147	0.0029	9.10E-07	24.9	0.0001
rs1163238	10	104943993	G	A	0.61	0.0135	0.0028	6.10E-07	24.2	0.0001
rs7900191	10	119145774	C	T	0.60	0.0139	0.0028	8.00E-07	25.4	0.0001
rs11200159	10	123553392	C	A	0.35	0.0142	0.0028	4.70E-07	25	0.0001

rs3808964	10	125426627	G	T	0.37	-0.0157	0.0028	2.70E-08	31.6	0.0001
rs9664044	10	126710791	C	T	0.77	0.0169	0.0032	1.60E-07	28	0.0001
rs10830107	10	129304075	A	G	0.80	0.0149	0.0034	7.20E-06	19.2	0.0001
rs76518095	10	131149976	C	T	0.92	-0.0238	0.0051	4.20E-06	22.1	0.0001
rs12771973	10	133749294	G	A	0.75	0.0136	0.0031	1.30E-05	19.2	0.0001
rs10832648	11	16618307	C	A	0.80	0.0198	0.0034	3.20E-09	34.3	0.0001
rs10742179	11	27650524	A	G	0.26	0.0183	0.0031	1.50E-09	35.6	0.0001
rs4923541	11	28479535	C	T	0.49	-0.0124	0.0027	4.60E-06	21.4	0.0001
rs621421	11	30405914	T	C	0.62	-0.0177	0.0028	7.50E-11	40.8	0.0001
rs11032362	11	33759092	G	A	0.91	-0.0396	0.0047	4.80E-17	71.6	0.0003
rs7111582	11	43893222	G	A	0.10	0.0278	0.0044	5.20E-10	39.3	0.0001
rs10838687	11	47312892	T	G	0.79	0.0166	0.0033	3.80E-07	25.3	0.0001
rs12808544	11	58373221	C	A	0.76	0.0208	0.0031	3.00E-11	43.5	0.0002
rs662094	11	66342691	G	A	0.50	-0.0136	0.0027	7.10E-07	25.5	0.0001
rs1278402	11	82972097	A	G	0.73	0.0110	0.0030	2.80E-04	13.1	4.8E-05
rs1508608	11	92893825	A	G	0.32	0.0133	0.0029	3.20E-06	21.4	0.0001
rs4121878	11	95120372	G	C	0.49	-0.0121	0.0027	4.80E-06	20.3	0.0001
rs17577073	11	99152801	A	C	0.57	0.0134	0.0027	1.30E-06	24.1	0.0001
rs2514214	11	113395329	A	G	0.27	0.0153	0.0030	5.90E-07	25.4	0.0001
rs4936290	11	114009255	A	C	0.65	-0.0177	0.0028	4.60E-10	39.2	0.0001
rs3867239	11	122093090	G	A	0.62	-0.0140	0.0028	8.70E-07	25.3	0.0001
rs74357745	11	122811822	A	G	0.88	0.0265	0.0041	8.70E-11	41.5	0.0002
rs7943634	11	126734319	C	T	0.69	0.0165	0.0029	1.40E-08	32	0.0001
rs11062167	12	364739	G	A	0.46	-0.0134	0.0027	4.50E-07	24.5	0.0001
rs1799464	12	16286082	A	G	0.29	-0.0135	0.0030	5.60E-06	20.8	0.0001
rs12298405	12	17015267	C	T	0.67	0.0159	0.0029	2.70E-08	30.6	0.0001
rs2433634	12	23060363	A	C	0.72	-0.0153	0.0030	3.30E-07	26.3	0.0001
rs11611435	12	24089322	T	C	0.55	0.0157	0.0027	8.60E-09	33.9	0.0001
rs13377754	12	34051765	T	C	0.61	0.0297	0.0028	6.90E-27	116.1	0.0004
rs247929	12	46294908	G	C	0.49	-0.0177	0.0027	6.20E-11	43.3	0.0002
rs7975791	12	49413486	C	T	0.96	-0.0346	0.0069	2.90E-07	25.1	0.0001
rs7299922	12	54702519	A	G	0.63	0.0161	0.0028	2.50E-09	33.3	0.0001
rs10877962	12	63520912	C	T	0.59	-0.0145	0.0027	8.20E-08	28.2	0.0001
rs711098	12	77976559	A	C	0.40	0.0096	0.0027	4.00E-04	12.3	4.5E-05
rs10777221	12	90441215	T	C	0.60	-0.0190	0.0027	4.90E-12	47.9	0.0002
rs7304278	12	106989915	A	G	0.27	-0.0203	0.0030	2.20E-11	44.9	0.0002
rs7298532	12	112510404	T	C	0.72	0.0164	0.0030	2.70E-08	30.3	0.0001
rs3955311	12	114343818	C	T	0.85	-0.0168	0.0038	6.90E-06	19.8	0.0001
rs80097534	12	121029604	G	T	0.90	0.0281	0.0046	9.30E-10	38.1	0.0001
rs9597241	13	56281271	A	C	0.81	0.0214	0.0035	7.50E-10	38.3	0.0001
rs9571526	13	66590868	T	G	0.77	-0.0134	0.0032	1.60E-05	17.7	0.0001
rs2593487	13	69903058	G	A	0.66	0.0148	0.0028	2.50E-07	27.3	0.0001

rs495593	13	72919800	G	A	0.26	-0.0129	0.0031	3.10E-05	17.5	0.0001
rs45597035	13	73649152	A	G	0.65	-0.0130	0.0028	3.40E-06	20.8	0.0001
rs9573980	13	77590741	A	G	0.97	0.0727	0.0075	5.70E-22	95.2	0.0003
rs1886205	13	94062095	C	A	0.24	-0.0194	0.0032	4.90E-10	37.8	0.0001
rs9558942	13	107700218	T	C	0.67	-0.0099	0.0029	7.20E-04	11.8	4.3E-05
rs3815983	13	109779906	C	T	0.64	0.0148	0.0028	1.50E-07	27.5	0.0001
rs1163628	13	112226420	A	C	0.86	-0.0188	0.0038	1.50E-06	24	0.0001
rs61990287	14	42069889	C	A	0.72	-0.0138	0.0030	3.00E-06	21.3	0.0001
rs2878172	14	55373670	A	G	0.57	-0.0104	0.0027	9.40E-05	14.6	0.0001
rs962961	14	57281154	C	T	0.67	0.0159	0.0029	2.00E-08	30.7	0.0001
rs6573308	14	60806976	C	T	0.61	-0.0119	0.0028	2.00E-05	18.4	0.0001
rs7143933	14	62460219	T	G	0.26	0.0114	0.0031	1.20E-04	13.9	0.0001
rs2978382	14	64769074	T	C	0.59	0.0140	0.0027	6.10E-07	26.1	0.0001
rs4903203	14	74660508	A	G	0.32	0.0145	0.0029	1.70E-07	25.4	0.0001
rs4550384	14	85350142	T	G	0.75	0.0157	0.0031	7.70E-07	25.2	0.0001
rs710284	14	98532540	T	C	0.58	0.0119	0.0027	1.70E-05	19	0.0001
rs11845599	14	101016824	A	G	0.64	-0.0153	0.0028	5.60E-08	29.4	0.0001
rs59986227	15	48009263	C	G	0.74	-0.0178	0.0031	8.30E-09	33.1	0.0001
rs12442008	15	53725112	C	T	0.75	-0.0143	0.0031	3.00E-06	21.1	0.0001
rs4775086	15	58969292	G	A	0.76	0.0138	0.0032	1.60E-05	18.9	0.0001
rs12442674	15	96907819	A	C	0.72	0.0113	0.0030	1.40E-04	14	0.0001
rs1873958	15	101147726	G	A	0.59	-0.0141	0.0028	1.30E-07	26.3	0.0001
rs72773411	16	728514	G	A	0.84	-0.0156	0.0037	2.30E-05	17.9	0.0001
rs12445235	16	8195278	G	C	0.59	0.0127	0.0027	6.80E-06	21.6	0.0001
rs2304467	16	8988777	C	G	0.60	-0.0141	0.0028	2.20E-07	26.1	0.0001
rs11641239	16	23124193	C	T	0.71	-0.0172	0.0030	1.10E-08	33.8	0.0001
rs7203707	16	24518569	C	A	0.48	0.0165	0.0027	7.60E-10	37.2	0.0001
rs4785296	16	49467234	G	C	0.77	-0.0152	0.0032	1.30E-06	22.7	0.0001
rs12927162	16	52684916	A	G	0.72	0.0286	0.0030	6.80E-22	91.2	0.0003
rs1421085	16	53800954	T	C	0.60	-0.0276	0.0027	1.50E-23	101.1	0.0004
rs2550298	16	56367969	C	T	0.62	0.0240	0.0028	3.60E-18	74.7	0.0003
rs8044054	16	60628436	C	T	0.61	-0.0192	0.0028	1.50E-12	48.6	0.0002
rs72790386	16	68136932	G	T	0.97	-0.0312	0.0075	1.70E-05	17.3	0.0001
rs17604349	16	72210865	G	A	0.82	0.0257	0.0035	1.70E-13	53.7	0.0002
rs1061032	17	8064083	T	G	0.09	0.0388	0.0048	2.00E-16	65.9	0.0002
rs11545787	17	17398278	G	A	0.75	0.0238	0.0031	4.90E-14	57.6	0.0002
rs12950382	17	30603994	A	G	0.72	0.0126	0.0030	3.80E-05	17.5	0.0001
rs4365329	17	31625887	A	T	0.54	-0.0108	0.0027	3.20E-05	16	0.0001
rs2011528	17	33980566	C	T	0.17	0.0207	0.0036	5.30E-09	33.2	0.0001
rs3760381	17	43047083	G	A	0.75	-0.0150	0.0031	9.30E-07	23.3	0.0001
rs12600452	17	45054564	G	A	0.79	-0.0146	0.0033	9.00E-06	19.2	0.0001
rs12051	17	46103760	A	G	0.61	-0.0146	0.0028	5.00E-08	27.8	0.0001

rs55846845	17	50092201	G	A	0.48	0.0160	0.0027	2.40E-09	35.3	0.0001
rs72829706	17	54173733	A	G	0.96	0.0407	0.0069	5.10E-09	34.5	0.0001
rs8072058	17	55734198	T	A	0.22	0.0142	0.0033	1.90E-05	19	0.0001
rs412000	17	56709058	G	C	0.44	0.0130	0.0027	1.90E-06	23	0.0001
rs58681483	17	57934654	A	G	0.92	0.0180	0.0050	3.70E-04	12.8	4.6E-05
rs72841368	17	61391114	A	T	0.81	-0.0198	0.0034	8.70E-09	32.9	0.0001
rs2916148	17	65482109	G	A	0.54	-0.0162	0.0027	3.50E-09	35.2	0.0001
rs2580160	18	1816036	A	G	0.55	0.0153	0.0027	1.70E-08	31.5	0.0001
rs62082402	18	5186566	G	T	0.81	-0.0256	0.0034	1.60E-14	56	0.0002
rs1788784	18	21159630	A	G	0.34	-0.0082	0.0028	1.70E-03	8.4	3.0E-05
rs1013987	18	22630836	T	C	0.41	-0.0183	0.0027	1.50E-11	44.3	0.0002
rs4419127	18	31663654	A	G	0.66	0.0218	0.0029	1.30E-14	58.3	0.0002
rs9950528	18	35762461	A	G	0.65	-0.0133	0.0029	3.10E-06	21.6	0.0001
rs12969848	18	38152835	C	T	0.47	-0.0219	0.0027	1.30E-15	65.4	0.0002
rs9956387	18	44773382	A	T	0.50	-0.0133	0.0027	5.00E-07	24.3	0.0001
rs4800998	18	53429655	T	A	0.81	-0.0155	0.0035	7.80E-06	20	0.0001
rs9964420	18	56824041	C	A	0.70	0.0285	0.0029	8.60E-22	94.1	0.0003
rs11152350	18	60240352	A	C	0.47	-0.0163	0.0027	1.30E-09	36.6	0.0001
rs34329963	18	64526233	C	T	0.89	0.0182	0.0042	1.20E-05	18.4	0.0001
rs1025601	18	73056278	C	T	0.62	0.0151	0.0028	1.30E-07	28.9	0.0001
rs10402849	19	2695661	C	T	0.80	-0.0193	0.0034	8.90E-09	32.9	0.0001
rs36055559	19	5799433	G	A	0.83	0.0211	0.0037	1.50E-08	32.1	0.0001
rs7248205	19	10770305	C	T	0.40	-0.0149	0.0028	3.80E-08	29.3	0.0001
rs9636202	19	18449238	G	A	0.73	0.0184	0.0031	7.50E-10	36.2	0.0001
rs73026775	19	31052954	G	A	0.87	0.0180	0.0042	1.20E-05	18.7	0.0001
rs4804951	19	31673388	A	G	0.33	0.0153	0.0029	1.80E-07	28.5	0.0001
rs58876439	19	42600984	G	A	0.93	-0.0288	0.0053	7.00E-08	29.5	0.0001
rs11670534	19	47003906	C	T	0.83	0.0210	0.0036	8.50E-09	33.4	0.0001
rs6131805	20	16222093	T	G	0.40	0.0156	0.0028	1.50E-08	31.2	0.0001
rs6131942	20	17348608	A	G	0.42	-0.0175	0.0027	9.40E-11	41.2	0.0001
rs1474754	20	20077178	A	G	0.27	-0.0129	0.0030	2.60E-05	18.1	0.0001
rs6047481	20	21539564	A	T	0.67	0.0140	0.0029	1.40E-06	23.5	0.0001
rs1737893	20	31051699	C	T	0.62	0.0142	0.0028	3.50E-07	25.8	0.0001
rs2072727	20	43538733	T	C	0.44	0.0164	0.0027	8.50E-10	36.3	0.0001
rs57236847	20	44668401	C	G	0.60	0.0113	0.0028	5.30E-05	16.9	0.0001
rs695459	22	28848278	C	T	0.61	0.0141	0.0028	3.20E-07	26	0.0001
rs28459838	22	35846168	T	C	0.23	0.0151	0.0032	1.90E-06	22.5	0.0001
rs139911	22	40704052	C	T	0.42	0.0234	0.0027	2.30E-17	73.3	0.0003
rs6007594	22	45728370	G	A	0.74	0.0161	0.0031	1.60E-07	27.7	0.0001

*The P-value is in the set of UK Biobank chronotype GWAS summary statistics.

Table 3. Associations of single nucleotide polymorphisms for sleep duration. Chr, chromosome; EA, effect allele; EAF, effect allele frequency; NEA, non-effect allele.

SNP	Chr	Position	EA	NEA	EAF	Beta*	SE*	P-value	F-statistic	R^2
rs915416	1	34731984	C	G	0.29	0.0193	0.0025	9.90E-15	59.6	0.0002
rs269054	1	57864304	T	A	0.58	-0.0136	0.0023	2.10E-09	35.4	0.0001
rs61796569	1	66476437	C	T	0.73	-0.0154	0.0026	1.50E-09	36.3	0.0001
rs12567114	1	98527951	G	A	0.72	-0.0148	0.0025	4.30E-09	34.1	0.0001
rs62120041	2	9185564	T	C	0.93	0.0261	0.0046	9.60E-09	32.6	0.0001
rs374153	2	40382712	C	T	0.16	0.0176	0.0031	9.10E-09	32.2	0.0001
rs75539574	2	58871658	A	C	0.91	-0.0362	0.0041	6.90E-19	79.5	0.0002
rs72804080	2	59358659	A	G	0.85	-0.0178	0.0032	2.90E-08	31	0.0001
rs7556815	2	114085785	G	A	0.78	-0.0407	0.0027	1.30E-49	220.9	0.0006
rs12611523	2	139195328	A	G	0.55	0.0126	0.0023	3.10E-08	30.8	0.0001
rs4128364	2	147612734	T	C	0.66	-0.0146	0.0024	1.40E-09	37.3	0.0001
rs4538155	2	157040773	C	T	0.35	-0.0130	0.0024	3.60E-08	29.9	0.0001
rs11885663	2	166944004	C	T	0.75	-0.0162	0.0026	8.60E-10	38.4	0.0001
rs10173260	2	210377845	T	C	0.39	-0.0128	0.0023	2.90E-08	30.8	0.0001
rs112230981	3	55879269	A	G	0.95	0.0315	0.0052	2.20E-09	36.4	0.0001
rs17732997	3	70470834	C	G	0.57	0.0129	0.0023	1.20E-08	32	0.0001
rs7644809	3	107564459	T	C	0.42	0.0131	0.0023	1.60E-08	32.2	0.0001
rs13088093	3	135838598	T	G	0.66	-0.0163	0.0024	7.00E-12	45.9	0.0001
rs2192528	4	18327896	A	G	0.48	0.0134	0.0023	2.70E-09	34.7	0.0001
rs17427571	4	82254908	A	G	0.68	0.0138	0.0024	1.30E-08	32.2	0.0001
rs35531607	4	92533225	T	C	0.53	-0.0128	0.0023	1.50E-08	31.9	0.0001
rs13109404	4	102896591	T	G	0.93	0.0312	0.0044	1.40E-12	50.1	0.0001
rs365663	5	1428883	A	G	0.55	0.0146	0.0023	1.00E-10	41.2	0.0001
rs460692	5	3126584	C	T	0.14	0.0211	0.0033	3.60E-10	39.9	0.0001
rs56372231	5	102321905	C	T	0.67	-0.0169	0.0024	2.20E-12	49.9	0.0001
rs11567976	5	137654218	C	T	0.43	-0.0128	0.0023	2.10E-08	31.4	0.0001
rs151014368	5	176751059	G	A	0.79	-0.0161	0.0028	9.10E-09	32.6	0.0001
rs34556183	6	28584775	A	G	0.72	0.0169	0.0025	2.30E-11	45	0.0001
rs80193650	6	33464363	A	G	0.84	-0.0168	0.0031	4.10E-08	30.1	0.0001
rs113113059	6	43160375	T	C	0.78	0.0161	0.0027	8.40E-09	34.8	0.0001
rs9382445	6	54937974	T	C	0.62	0.0145	0.0023	4.80E-10	38.8	0.0001
rs2231265	6	89790201	A	G	0.23	-0.0150	0.0027	2.70E-08	30.7	0.0001
rs9345234	6	93162639	A	C	0.42	-0.0130	0.0023	1.80E-08	32	0.0001
rs34731055	7	2106928	C	T	0.82	-0.0195	0.0029	3.70E-11	43.6	0.0001
rs2079070	7	114126432	C	G	0.26	0.0175	0.0026	7.50E-12	46.8	0.0001
rs7806045	7	132610266	T	C	0.75	0.0148	0.0026	1.40E-08	31.7	0.0001
rs330088	8	9149746	T	C	0.45	-0.0145	0.0023	2.70E-10	40.4	0.0001
rs73219758	8	14279446	G	A	0.71	0.0164	0.0025	5.60E-11	43.2	0.0001
rs10973207	9	37100525	G	T	0.84	-0.0204	0.0031	6.00E-11	42.8	0.0001

rs1776776	9	140497072	T	C	0.87	0.0200	0.0034	4.90E-09	34.3	0.0001
rs12246842	10	21830580	A	G	0.46	0.0134	0.0023	3.90E-09	34.7	0.0001
rs10761674	10	64618340	C	T	0.48	0.0123	0.0023	4.20E-08	29.6	0.0001
rs11190970	10	103128332	G	A	0.80	0.0154	0.0028	4.60E-08	29.7	0.0001
rs7915425	10	125016501	T	C	0.17	0.0191	0.0030	2.00E-10	40.7	0.0001
rs1517572	11	28829882	A	C	0.42	-0.0146	0.0023	1.50E-10	40.7	0.0001
rs4592416	11	43800474	A	G	0.54	-0.0147	0.0023	9.30E-11	41.8	0.0001
rs11602180	11	48162453	C	T	0.84	0.0182	0.0031	2.30E-09	35.4	0.0001
rs174560	11	61581764	T	C	0.69	-0.0136	0.0024	2.80E-08	31	0.0001
rs12791153	11	80685181	A	T	0.92	-0.0235	0.0042	1.90E-08	31.2	0.0001
rs1553132	11	88297740	A	G	0.74	-0.0145	0.0026	2.50E-08	31.5	0.0001
rs1939455	11	101520886	G	T	0.88	0.0204	0.0036	1.20E-08	32.9	0.0001
rs7115226	11	113408518	C	A	0.93	-0.0266	0.0044	1.70E-09	37.2	0.0001
rs1263056	11	116576415	A	G	0.52	0.0128	0.0023	2.00E-08	31.6	0.0001
rs7951019	11	118358027	T	G	0.97	-0.0369	0.0065	1.20E-08	32	0.0001
rs1057703	11	122830251	T	G	0.85	-0.0194	0.0032	1.10E-09	36.6	0.0001
rs34354917	12	38764559	C	A	0.71	0.0137	0.0025	3.90E-08	30.2	0.0001
rs4767550	12	117951150	A	G	0.59	-0.0143	0.0023	6.30E-10	38.3	0.0001
rs6575005	14	26954078	T	C	0.76	0.0156	0.0026	4.40E-09	34.7	0.0001
rs10483350	14	29816155	A	G	0.80	-0.0174	0.0029	1.50E-09	36.7	0.0001
rs61985058	14	60233841	C	T	0.86	-0.0186	0.0032	1.30E-08	33.2	0.0001
rs55658675	14	65554638	C	T	0.64	0.0131	0.0024	2.00E-08	30.8	0.0001
rs11621908	14	78495761	C	T	0.92	0.0241	0.0042	5.60E-09	33.5	0.0001
rs8038326	15	47989799	A	G	0.73	0.0159	0.0025	2.80E-10	39.3	0.0001
rs3095508	16	6550400	C	A	0.59	0.0154	0.0023	3.10E-11	44.4	0.0001
rs11643715	16	23909538	C	G	0.71	-0.0139	0.0025	3.20E-08	31	0.0001
rs9940646	16	53800629	C	G	0.58	0.0169	0.0023	1.20E-13	54.7	0.0001
rs8050478	16	56120461	G	A	0.50	0.0160	0.0023	1.70E-12	49.9	0.0001
rs7503199	17	8134275	C	T	0.73	0.0147	0.0026	1.00E-08	33.1	0.0001
rs205024	17	11227352	C	T	0.62	-0.0138	0.0023	3.90E-09	35.3	0.0001
rs1991556	17	44083402	G	A	0.77	0.0166	0.0027	1.00E-09	37	0.0001
rs9903973	17	50571227	C	T	0.47	0.0128	0.0023	2.60E-08	31.6	0.0001
rs12607679	18	53059748	T	C	0.74	0.0201	0.0026	8.30E-15	60.3	0.0002
rs10421649	19	9942262	T	A	0.44	-0.0133	0.0023	6.90E-09	33.6	0.0001
rs2072727	20	43538733	T	C	0.44	0.0132	0.0023	7.90E-09	33.6	0.0001

* Effect estimate and standard error of effect estimate for SNP on sleep duration per hour.

Table 4. Associations of single nucleotide polymorphisms for short sleep duration. Chr, chromosome; EA, effect allele; EAF, effect allele frequency; NEA, non-effect allele.

SNP	Chr	Position	EA	NEA	EAF	Beta	SE	P-value	F-statistic	R^2
rs7524118	1	34736052	T	C	0.29	-0.030	0.0065	4.90E-08	21.4	0.0004
rs2186122	1	66470206	A	T	0.44	-0.024	0.0060	4.80E-09	15.9	0.0003
rs12567114	1	98527951	G	A	0.72	0.036	0.0064	4.10E-09	31.3	0.0005
rs2820313	1	201870221	A	G	0.66	-0.031	0.0060	2.30E-09	26.9	0.0004
rs1380703	2	57941287	A	G	0.62	-0.035	0.0059	1.60E-11	34.7	0.0006
rs75539574	2	58871658	A	C	0.91	0.045	0.0108	8.40E-11	17.2	0.0003
rs2863957	2	114089551	C	A	0.78	0.054	0.0073	2.60E-18	54.7	0.001
rs2014830	3	50172397	C	T	0.70	0.030	0.0065	2.70E-08	21.4	0.0004
rs17005118	4	82288564	G	A	0.74	-0.030	0.0065	2.50E-09	21.4	0.0004
rs13107325	4	103188709	C	T	0.93	-0.075	0.0110	2.50E-13	46.5	0.0008
rs12518468	5	7249696	T	C	0.67	-0.031	0.0065	8.50E-09	23	0.0004
rs3776864	5	102327868	A	C	0.67	0.031	0.0065	1.70E-08	23	0.0004
rs4585442	5	135508381	A	G	0.69	-0.031	0.0060	8.10E-10	26.9	0.0004
rs12661667	6	41792545	C	T	0.74	-0.028	0.0065	2.80E-08	18.6	0.0003
rs9367621	6	55040290	T	A	0.43	0.024	0.0055	1.60E-08	19	0.0003
rs9321171	6	129848635	C	T	0.54	0.031	0.0060	4.20E-08	27	0.0005
rs11763750	7	2080114	G	A	0.81	0.035	0.0079	5.10E-09	19.4	0.0004
rs1229762	7	114218582	C	T	0.34	-0.037	0.0064	1.00E-12	33.1	0.0006
rs60882754	8	52886619	A	T	0.94	0.055	0.0122	1.80E-08	20.3	0.0003
rs1607227	11	28808617	G	T	0.70	0.031	0.0065	1.50E-09	22.9	0.0004
rs7939345	11	47980568	A	G	0.21	0.035	0.0074	4.00E-08	22.1	0.0004
rs17388803	15	48027204	A	C	0.89	-0.053	0.0098	6.50E-10	29.4	0.0005
rs59779556	16	56227965	T	G	0.55	0.025	0.0060	2.00E-08	17.3	0.0003
rs205024	17	11227352	C	T	0.62	0.031	0.0065	2.70E-08	22.9	0.0005
rs12963463	18	53099093	C	G	0.30	0.029	0.0065	1.90E-11	20	0.0004
rs5757675	22	39838892	G	T	0.26	0.034	0.0069	2.70E-09	23.9	0.0004

Table 5. Associations of single nucleotide polymorphisms for long sleep duration. Chr, chromosome; EA, effect allele; EAF, effect allele frequency; NEA, non-effect allele.

SNP	Chr	Position	EA	NEA	EAF	Beta	SE	P-value	F-statistic	R^2
rs7534398	1	7767464	T	A	0.80	-0.0469	0.0118	2.10E-08	15.7	0.0007
rs6737318	2	114083120	A	G	0.78	-0.0760	0.0110	3.40E-13	47.8	0.002
rs10899257	11	76415209	G	A	0.86	-0.0677	0.0130	4.60E-08	26.9	0.0011
rs75458655	11	118115331	C	T	0.98	-0.1848	0.0292	5.40E-12	40	0.0015
rs3751046	11	122828342	A	G	0.85	-0.0695	0.0135	2.00E-08	26.5	0.0012
rs17817288	16	53807764	A	G	0.52	0.0392	0.0094	8.90E-09	17.4	0.0008
rs17688916	17	43778680	T	A	0.80	0.0714	0.0125	1.10E-11	32.6	0.0017

Table 6. Associations of single nucleotide polymorphisms for daytime napping. Chr, chromosome; EA, effect allele; EAF, effect allele frequency; NEA, non-effect allele.

**SNP**	**Chr**	**Position**	**EA**	**NEA**	**EAF**	**Beta**	**SE**	**P-value**	**F-statistic**	**R^2**
rs2786547	1	33342304	C	T	0.82	0.0108	0.0016	1.00E-11	46	3.4E-05
rs12140153	1	62579891	G	T	0.90	0.0247	0.0021	2.40E-31	138.3	0.0001
rs2893323	1	95783037	G	A	0.65	-0.0069	0.0013	3.30E-08	30	2.2E-05
rs1931175	1	96928273	C	G	0.62	-0.0078	0.0012	4.60E-10	38.7	2.8E-05
rs1843815	1	98589715	A	T	0.45	-0.0073	0.0012	3.30E-09	35.7	2.6E-05
rs12031519	1	162876200	A	G	0.89	0.0113	0.0019	5.50E-09	33.7	2.5E-05
rs35039375	1	171516863	A	G	0.91	-0.0136	0.0021	8.70E-11	41.8	3.0E-05
rs2250377	1	201860626	A	G	0.34	0.0132	0.0013	1.40E-24	105.7	7.8E-05
rs13033444	2	23611438	A	G	0.72	-0.0097	0.0014	3.60E-13	52.1	3.8E-05
rs56180058	2	25337155	C	T	0.84	0.0094	0.0017	1.30E-08	32.3	2.4E-05
rs9309116	2	44557919	C	T	0.35	-0.0074	0.0013	3.90E-09	33.6	2.5E-05
rs13023587	2	49413860	C	G	0.49	0.0074	0.0012	6.50E-10	37.8	2.8E-05
rs350785	2	52934600	T	C	0.12	0.0126	0.0019	1.50E-11	43.9	3.3E-05
rs17049683	2	58905715	A	G	0.67	-0.0080	0.0013	4.10E-10	38.0	2.8E-05
rs11125776	2	59478517	T	G	0.85	0.0117	0.0017	1.50E-11	45.6	3.4E-05
rs12614085	2	151501545	C	T	0.09	0.0123	0.0022	2.00E-08	32.4	2.4E-05
rs62189006	2	162573586	A	G	0.91	0.0121	0.0021	8.30E-09	33.2	2.5E-05
rs80163246	2	164574344	T	C	0.88	-0.0119	0.0019	1.40E-10	40.0	2.9E-05
rs35144585	2	169092428	T	A	0.87	0.0103	0.0018	1.20E-08	32.0	2.4E-05
rs3732085	2	206831860	C	A	0.64	0.0072	0.0013	1.50E-08	32.4	2.4E-05
rs7422655	2	208941004	C	T	0.26	0.0078	0.0014	1.10E-08	32.3	2.4E-05
rs77154532	3	19377311	A	G	0.64	0.0077	0.0013	1.40E-09	36.7	2.7E-05
rs936944	3	44385814	G	A	0.16	0.0091	0.0016	3.20E-08	30.9	2.3E-05
rs76824303	3	62459819	A	C	0.90	0.0118	0.0021	1.70E-08	32.2	2.5E-05
rs9883093	3	82774831	T	G	0.37	-0.0073	0.0013	4.50E-09	34.1	2.5E-05
rs1601440	3	84665393	C	T	0.28	0.0091	0.0014	1.90E-11	45.3	3.3E-05
rs2699869	3	133032892	A	C	0.45	0.0068	0.0012	3.10E-08	31.0	2.3E-05
rs253666	3	138115770	G	A	0.24	-0.0081	0.0014	1.80E-08	32.3	2.4E-05
rs1001817	3	183995341	C	T	0.51	0.0078	0.0012	1.70E-10	40.8	3.0E-05
rs9998136	4	79550425	C	G	0.25	-0.0088	0.0014	4.40E-10	39.5	2.9E-05
rs13150944	4	85408000	A	G	0.31	-0.0086	0.0013	6.80E-11	42.3	3.2E-05
rs4356873	4	164216739	T	C	0.76	-0.0078	0.0014	4.50E-08	29.8	2.2E-05
rs4692709	4	170228549	C	T	0.45	0.0072	0.0012	2.40E-09	34.9	2.6E-05
rs12657723	5	62760828	C	T	0.68	-0.0082	0.0013	2.00E-10	39.7	2.9E-05
rs6452787	5	87712831	A	G	0.54	0.0077	0.0012	2.00E-10	40.3	3.0E-05
rs2943023	5	89597733	C	T	0.58	0.0071	0.0012	7.40E-09	33.3	2.5E-05
rs2195272	5	102295504	G	T	0.67	-0.0101	0.0013	6.70E-15	61.5	4.5E-05
rs2431108	5	103947968	T	C	0.67	-0.0130	0.0013	7.70E-24	101.2	7.4E-05
rs4357022	5	106311039	T	G	0.54	-0.0068	0.0012	1.40E-08	31.5	2.3E-05

rs388016	5	106861892	A	G	0.67	0.0074	0.0013	6.10E-09	32.9	2.4E-05
rs2099810	5	112030173	A	G	0.50	0.0077	0.0012	2.80E-10	40.1	3.0E-05
rs10875606	5	143769614	C	A	0.32	0.0074	0.0013	1.30E-08	31.8	2.4E-05
rs10875622	5	146693062	G	A	0.42	-0.0105	0.0012	1.30E-17	72.5	5.3E-05
rs73817091	5	159042652	C	T	0.96	-0.0165	0.0030	3.80E-08	29.9	2.2E-05
rs11967137	6	28199764	A	G	0.81	0.0093	0.0015	1.70E-09	36.6	2.7E-05
rs34262487	6	36224315	C	A	0.93	0.0145	0.0024	1.20E-09	38.0	2.8E-05
rs6919087	6	37699156	T	G	0.69	0.0108	0.0013	1.00E-16	67.6	5.0E-05
rs4236060	6	38470087	C	T	0.73	-0.0088	0.0014	8.80E-11	41.2	3.1E-05
rs2653349	6	55142337	A	G	0.21	0.0166	0.0015	3.40E-29	126.0	9.3E-05
rs2143792	6	62182204	G	A	0.57	0.0071	0.0012	9.10E-09	33.4	2.5E-05
rs9389556	6	100079774	C	G	0.74	-0.0084	0.0014	9.70E-10	37.0	2.7E-05
rs614987	6	124920871	A	C	0.39	-0.0110	0.0012	7.50E-19	77.8	5.8E-05
rs140506252	6	155129280	A	T	0.98	0.0227	0.0041	4.30E-08	30.9	2.3E-05
rs9460110	6	170621249	T	C	0.63	-0.0074	0.0013	8.40E-09	34.2	2.5E-05
rs35851551	7	31330785	A	G	0.90	0.0112	0.0020	3.50E-08	30.5	2.3E-05
rs10257273	7	99188589	A	T	0.85	0.0107	0.0017	8.90E-10	39.7	2.9E-05
rs13263535	8	1175131	G	T	0.53	0.0069	0.0012	1.60E-08	31.9	2.4E-05
rs351776	8	28191306	A	C	0.45	-0.0076	0.0012	8.40E-10	38.3	2.8E-05
rs2059639	8	67381858	C	T	0.48	0.0067	0.0012	2.40E-08	30.1	2.2E-05
rs285815	8	106108303	T	A	0.46	0.0075	0.0012	5.80E-10	38.1	2.8E-05
rs7814873	8	142213997	C	T	0.38	0.0072	0.0013	1.50E-08	32.5	2.5E-05
rs10811438	9	20962282	G	C	0.61	0.0072	0.0012	3.20E-09	33.7	2.5E-05
rs62560863	9	34081331	C	T	0.90	-0.0113	0.0020	2.50E-08	31.3	2.3E-05
rs17502738	9	37441650	T	C	0.80	0.0087	0.0015	2.00E-08	32.4	2.4E-05
rs1415218	9	73504992	T	C	0.28	0.0082	0.0014	1.20E-09	36.8	2.7E-05
rs13284688	9	81727018	T	C	0.79	-0.0151	0.0015	1.70E-23	101.1	7.4E-05
rs971415	9	108846445	A	G	0.88	0.0111	0.0019	1.20E-09	35.7	2.6E-05
rs4604518	9	120555845	G	A	0.55	0.0069	0.0012	1.40E-08	32.1	2.4E-05
rs12346996	9	131840856	T	C	0.27	0.0080	0.0014	5.00E-09	34.0	2.5E-05
rs11258652	10	13868855	C	A	0.76	0.0104	0.0014	3.70E-13	53.0	3.9E-05
rs224111	10	64552010	G	A	0.61	0.0080	0.0012	1.60E-10	40.9	3.0E-05
rs10835420	11	28866669	T	A	0.75	0.0089	0.0014	1.70E-10	40.3	3.0E-05
rs174541	11	61565908	T	C	0.64	-0.0099	0.0013	4.40E-15	61.2	4.5E-05
rs271057	11	92769748	C	T	0.25	0.0087	0.0014	6.50E-10	38.1	2.8E-05
rs11224896	11	101479583	T	C	0.89	0.0112	0.0019	1.10E-08	33.1	2.4E-05
rs2417268	12	13493906	A	T	0.56	-0.0075	0.0012	1.20E-09	37.1	2.8E-05
rs35011311	12	38616581	G	T	0.73	0.0091	0.0014	4.40E-11	43.2	3.2E-05
rs60222088	12	108334477	C	A	0.85	0.0112	0.0017	6.40E-11	42.5	3.2E-05
rs11615756	12	117942076	C	T	0.60	-0.0183	0.0012	1.40E-49	217.4	0.0002
rs2769916	13	107820389	G	A	0.31	-0.0088	0.0013	1.70E-11	44.4	3.3E-05
rs4983329	14	29218473	A	C	0.56	0.0077	0.0012	3.10E-10	39.5	2.9E-05

rs10149986	14	29721044	T	G	0.81	-0.0109	0.0016	4.40E-12	48.8	3.6E-05
rs2370926	14	79602542	T	C	0.63	0.0081	0.0013	1.70E-10	41.4	3.1E-05
rs11071755	15	63793936	G	A	0.57	0.0071	0.0012	5.20E-09	33.6	2.5E-05
rs17158413	15	83235408	G	A	0.76	-0.0093	0.0014	4.40E-11	41.9	3.1E-05
rs10152428	15	93510243	G	C	0.27	-0.0078	0.0014	1.10E-08	32.5	2.4E-05
rs60920123	16	10134637	G	A	0.57	0.0076	0.0012	4.50E-10	38.7	2.9E-05
rs9939355	16	23837048	C	T	0.44	0.0073	0.0012	4.50E-09	35.2	2.6E-05
rs3986805	16	28350059	A	G	0.59	-0.0072	0.0012	8.70E-09	33.8	2.5E-05
rs1592544	16	56129244	C	T	0.50	0.0087	0.0012	8.50E-13	50.7	3.8E-05
rs528301822	16	69403012	A	T	0.71	-0.0080	0.0013	2.80E-09	35.8	2.6E-05
rs12451365	17	35595368	T	C	0.80	-0.0106	0.0015	1.50E-12	49.9	3.7E-05
rs385199	17	43686419	A	C	0.77	0.0209	0.0014	7.50E-47	208.1	0.0002
rs112520848	17	64308310	G	C	0.61	-0.0070	0.0013	1.80E-08	31.5	2.3E-05
rs3935190	17	79084367	G	A	0.46	-0.0080	0.0012	5.40E-11	42.9	3.2E-05
rs962247	18	23084118	G	A	0.52	0.0080	0.0012	5.70E-11	42.3	3.2E-05
rs1941182	18	25569743	C	A	0.58	-0.0068	0.0012	4.10E-08	29.9	2.2E-05
rs34728579	18	31610848	T	C	0.80	-0.0084	0.0015	3.70E-08	30.5	2.3E-05
rs2861805	18	36105007	A	G	0.54	0.0093	0.0012	2.00E-14	58.2	4.3E-05
rs9965170	18	44788274	G	A	0.58	0.0136	0.0012	7.80E-29	123.2	9.1E-05
rs17265513	20	39832628	T	C	0.80	-0.0091	0.0015	2.00E-09	36.1	2.7E-05
rs910187	20	45841052	G	A	0.63	0.0073	0.0013	4.90E-09	33.9	2.5E-05
rs3810484	20	62194103	A	G	0.56	0.0068	0.0012	2.20E-08	31.3	2.3E-05
rs1883048	21	47397586	T	C	0.48	-0.0079	0.0012	1.80E-10	41.4	3.1E-05
rs2284015	22	37096573	C	G	0.74	-0.0077	0.0014	4.10E-08	30.9	2.3E-05

Table 7. Associations of single nucleotide polymorphisms for daytime sleepiness. Chr, chromosome; EA, effect allele; EAF, effect allele frequency; NEA, non-effect allele.

**SNP**	**Chr**	**Position**	**EA**	**NEA**	**EAF**	**Beta**	**SE**	**P-value**	**F-statistic**	**R^2**
rs2787120	1	33306297	A	G	0.83	0.0078	0.0014	2.00E-08	31.9	1.7E-05
rs12140153	1	62579891	G	T	0.90	0.0166	0.0018	2.80E-20	85.2	4.7E-05
rs17131124	1	91127548	C	G	0.91	-0.0112	0.0018	1.70E-09	37.0	2.0E-05
rs57746981	1	201885234	C	T	0.64	0.0068	0.0011	2.20E-10	40.1	2.1E-05
rs825127	1	223506788	T	G	0.53	0.0059	0.0010	9.50E-09	32.8	1.7E-05
rs4665972	2	27598097	T	C	0.39	0.0066	0.0011	3.90E-10	39.2	2.1E-05
rs7598712	2	46660452	G	T	0.56	0.0058	0.0010	2.20E-08	30.9	1.7E-05
rs6741951	2	58959112	G	A	0.71	0.0068	0.0011	2.70E-09	35.7	1.9E-05
rs11123962	2	104157011	T	G	0.55	-0.0080	0.0010	7.50E-15	60.5	3.2E-05
rs9712275	2	198907143	C	T	0.49	-0.0059	0.0010	1.30E-08	32.5	1.7E-05
rs7607363	2	213402705	A	G	0.56	-0.0060	0.0010	8.00E-09	33.7	1.8E-05
rs13010456	2	236792801	A	G	0.59	0.0077	0.0011	2.10E-13	54.2	2.9E-05
rs13097760	3	82823561	A	C	0.64	-0.0060	0.0011	3.20E-08	30.9	1.7E-05

rs960986	3	85519305	C	T	0.64	0.0072	0.0011	1.50E-11	45.0	2.4E-05
rs843372	3	183996213	C	T	0.23	0.0082	0.0012	2.20E-11	44.3	2.4E-05
rs11942333	4	46389486	G	A	0.68	-0.0061	0.0011	3.80E-08	30.1	1.6E-05
rs13135092	4	103198082	A	G	0.92	-0.0103	0.0019	3.10E-08	30.3	1.6E-05
rs6897863	5	92512481	A	C	0.58	0.0065	0.0010	7.60E-10	38.0	2.0E-05
rs12153518	5	138501494	A	G	0.47	0.0067	0.0010	6.80E-11	42.2	2.2E-05
rs6923811	6	27289776	T	C	0.68	0.0068	0.0011	9.10E-10	37.6	2.0E-05
rs55960940	6	38153146	T	C	0.82	0.0076	0.0014	2.00E-08	31.8	1.7E-05
rs3122170	6	55058998	C	A	0.23	0.0095	0.0012	5.60E-15	60.0	3.2E-05
rs641498	6	124911565	A	G	0.39	-0.0054	0.0011	2.70E-07	26.4	1.4E-05
rs62519825	8	65479707	T	C	0.89	-0.0095	0.0016	3.80E-09	33.9	1.8E-05
rs285793	8	106087862	G	A	0.46	0.0068	0.0010	7.90E-11	42.7	2.3E-05
rs7837226	8	131235895	A	G	0.47	-0.0057	0.0010	2.00E-08	30.9	1.6E-05
rs55818482	9	81744922	T	C	0.78	-0.0097	0.0013	1.40E-14	59.7	3.2E-05
rs1566362	9	128163132	T	C	0.63	0.0063	0.0011	3.80E-09	35.0	1.9E-05
rs7476897	10	92416402	G	A	0.68	0.0074	0.0011	2.70E-11	45.5	2.4E-05
rs12253139	10	128899947	T	C	0.88	-0.0083	0.0016	1.20E-07	28.0	1.5E-05
rs4765939	12	2582397	G	C	0.58	-0.0063	0.0010	2.00E-09	36.0	1.9E-05
rs1846644	12	117938380	T	C	0.59	-0.0114	0.0010	2.50E-27	117.4	6.2E-05
rs7982022	13	54049003	G	A	0.56	-0.0056	0.0011	1.60E-07	27.7	1.5E-05
rs8015449	14	82161860	A	G	0.54	0.0062	0.0010	1.90E-09	35.8	1.9E-05
rs2472297	15	75027880	C	T	0.73	0.0058	0.0012	6.80E-07	39.3	2.1E-05
rs17356118	15	83237899	A	G	0.77	-0.0077	0.0012	2.60E-10	29.2	1.5E-05
rs7162082	15	83896608	C	T	0.80	0.0069	0.0013	6.60E-08	24.9	1.3E-05
rs886114	16	23865986	C	T	0.36	0.0060	0.0011	1.90E-08	31.6	1.7E-05
rs11078398	17	17697099	G	A	0.74	0.0077	0.0012	7.10E-10	38.1	2.2E-05
rs2048522	18	44800515	A	T	0.57	0.0058	0.0011	3.50E-08	30.7	1.7E-05

Table 8. Associations of single nucleotide polymorphisms for insomnia. Chr, chromosome; EA, effect allele; EAF, effect allele frequency; NEA, non-effect allele.

SNP	Chr	Position	EA	NEA	EAF	Beta	SE	P-value	F-statistic	R^2
rs4751	1	1686040	G	T	0.58	-0.0077	0.0014	1.60E-08	30.6	3.0E-05
rs12405761	1	57850914	A	C	0.57	0.0092	0.0014	2.60E-11	43.6	4.0E-05
rs2613503	1	72839774	C	A	0.20	-0.0088	0.0017	2.80E-07	25.9	2.0E-05
rs11804386	1	87738947	G	A	0.67	-0.0081	0.0015	2.60E-08	30.3	3.0E-05
rs11184946	1	107185225	C	T	0.58	-0.0088	0.0014	2.90E-10	39.4	4.0E-05
rs6664467	1	151738403	G	A	0.86	0.0113	0.0020	4.50E-08	31.2	3.0E-05
rs2644128	1	201793440	C	G	0.45	-0.0097	0.0014	1.00E-12	49.3	5.0E-05
rs35881094	2	58922921	T	G	0.57	-0.0111	0.0014	3.00E-15	62.2	6.0E-05
rs2192338	2	59833168	G	C	0.22	-0.0088	0.0017	1.20E-07	28	3.0E-05
rs113851554	2	66750564	G	T	0.94	-0.0414	0.0031	1.30E-41	181.2	1.8E-04

rs62158170	2	114082175	A	G	0.79	0.0121	0.0017	5.70E-13	51.3	5.0E-05
rs4577309	2	191288833	A	G	0.47	0.0081	0.0014	3.70E-09	34.2	3.0E-05
rs4688760	3	49980596	C	T	0.31	-0.0108	0.0015	1.00E-12	51.6	5.0E-05
rs55946513	3	52599792	C	T	0.93	0.0124	0.0028	7.30E-06	19.8	2.0E-05
rs9845387	3	116425935	C	A	0.96	0.0197	0.0035	2.10E-08	31.5	3.0E-05
rs6785034	3	184781830	G	A	0.58	-0.0055	0.0014	9.60E-05	15.2	1.0E-05
rs1841625	4	91287477	A	G	0.57	-0.0077	0.0014	3.60E-08	30.4	3.0E-05
rs11097861	4	105330133	A	G	0.28	-0.0092	0.0015	1.70E-09	35.6	3.0E-05
rs1430205	5	87678585	C	T	0.54	-0.0077	0.0014	3.60E-08	30.6	3.0E-05
rs28061	5	102543878	A	G	0.69	0.0084	0.0015	2.10E-08	30.9	3.0E-05
rs1592757	5	103889998	G	C	0.64	-0.0090	0.0014	4.60E-10	39.2	4.0E-05
rs7711696	5	135486536	G	T	0.70	-0.0102	0.0015	9.90E-12	46	4.0E-05
rs10947690	6	37631768	A	G	0.74	-0.0087	0.0016	3.50E-08	30.5	3.0E-05
rs6932158	6	101246010	T	C	0.51	-0.0078	0.0014	2.80E-08	31.4	3.0E-05
rs314280	6	105400837	A	G	0.45	-0.0086	0.0014	3.50E-10	38.7	4.0E-05
rs3824081	7	1024581	T	C	0.48	0.0080	0.0014	1.10E-08	33.3	3.0E-05
rs302165	7	18323899	G	A	0.22	0.0079	0.0017	2.40E-06	22	2.0E-05
rs6593005	7	52584625	A	G	0.26	-0.0090	0.0016	8.60E-09	32.7	3.0E-05
rs10280045	7	114076394	C	G	0.43	-0.0091	0.0014	1.00E-10	41.5	4.0E-05
rs17151854	8	10236559	G	T	0.85	-0.0107	0.0019	2.40E-08	31	3.0E-05
rs11793831	9	23362311	G	T	0.58	0.0067	0.0014	1.50E-06	22.6	2.0E-05
rs11793074	9	23820070	A	G	0.85	0.0101	0.0019	2.30E-07	27	3.0E-05
rs10156602	9	96345328	A	G	0.64	0.0101	0.0014	3.40E-12	48.8	5.0E-05
rs2296580	10	104241683	G	T	0.70	0.0103	0.0015	8.70E-12	46.6	4.0E-05
rs10838708	11	47441513	G	A	0.54	0.0084	0.0014	2.50E-09	36	4.0E-05
rs324017	12	57487814	A	C	0.29	0.0097	0.0015	1.10E-10	40.6	4.0E-05
rs2956278	12	84698234	A	G	0.79	-0.0095	0.0017	1.30E-08	32	3.0E-05
rs68094047	12	109855201	C	T	0.75	-0.0094	0.0016	3.30E-09	34.6	3.0E-05
rs1923770	13	53786568	T	A	0.38	0.0100	0.0014	2.30E-12	49	5.0E-05
rs1031654	13	54382035	C	A	0.20	0.0103	0.0017	2.00E-09	35.4	3.0E-05
rs4886140	13	59833519	A	G	0.33	-0.0092	0.0015	3.10E-10	39	4.0E-05
rs2147141	13	112707954	C	G	0.46	-0.0076	0.0014	8.20E-08	29.4	3.0E-05
rs11635495	15	67804682	T	C	0.49	-0.0081	0.0014	6.80E-09	34.4	3.0E-05
rs4886860	15	74340336	G	C	0.23	0.0112	0.0016	6.10E-12	47.4	5.0E-05
rs17139246	16	6106260	T	C	0.61	-0.0078	0.0014	4.10E-08	29.6	3.0E-05
rs1544637	16	51484837	T	C	0.49	0.0077	0.0014	3.00E-08	30.6	3.0E-05
rs3104778	16	52633652	A	G	0.59	0.0078	0.0014	4.20E-08	30.6	3.0E-05
rs2062113	16	59476179	T	C	0.43	0.0089	0.0014	1.90E-10	40.2	4.0E-05
rs17669584	17	28899614	A	G	0.80	-0.0096	0.0018	3.60E-08	29.2	3.0E-05
rs11651809	17	43255681	C	G	0.70	-0.0122	0.0015	2.10E-15	64.3	6.0E-05
rs1942262	18	52873317	G	A	0.71	-0.0112	0.0015	1.10E-13	53.6	5.0E-05
rs11673344	19	37684966	A	G	0.62	-0.0087	0.0014	9.00E-10	36.9	4.0E-05

Table 9. Associations of single nucleotide polymorphisms for schizophrenia. Chr, chromosome; EA, effect allele; EAF, effect allele frequency; NEA, non-effect allele.

SNP	Chr	Position	EA	NEA	EAF	Beta	SE	P-value	F-statistic	R^2
rs4648845	1	2387101	T	C	0.48	0.0672	0.0119	1.74E-08	31.9	0.0023
rs1702294	1	98501984	C	T	0.78	0.1184	0.0138	1.03E-17	73.6	0.0047
rs11210892	1	44100084	A	G	0.65	-0.0678	0.0115	3.42E-09	34.8	0.0021
rs301797	1	8487323	A	C	0.32	0.0661	0.0116	1.20E-08	32.5	0.0019
rs1498232	1	30433951	C	T	0.70	-0.0720	0.0118	1.21E-09	37.2	0.0022
rs12062861	1	150017281	A	G	0.18	-0.0911	0.0149	9.66E-10	37.4	0.0024
rs1538774	1	243544827	G	C	0.72	0.0704	0.0126	2.32E-08	31.2	0.0020
rs35998080	1	73278615	T	G	0.45	0.0690	0.0112	6.95E-10	38.0	0.0024
rs77149735	1	243555105	A	G	0.02	0.2845	0.0485	4.40E-09	34.4	0.0025
rs2909457	2	162845855	A	G	0.57	-0.0597	0.0109	4.25E-08	30.0	0.0017
rs11685299	2	225391296	A	C	0.32	-0.0662	0.0117	1.49E-08	32.0	0.0019
rs11693094	2	185601420	T	C	0.43	-0.0736	0.0110	2.17E-11	44.8	0.0027
rs59979824	2	193848340	A	C	0.34	-0.0710	0.0119	2.73E-09	35.6	0.0022
rs6430095	2	146439945	A	G	0.21	0.0798	0.0145	3.40E-08	30.3	0.0021
rs11682175	2	57987593	C	T	0.44	0.0735	0.0109	1.58E-11	45.5	0.0027
rs6434928	2	198304577	A	G	0.70	-0.0787	0.0116	1.17E-11	46.0	0.0026
rs7601312	2	229320093	G	A	0.48	0.0590	0.0108	4.67E-08	29.8	0.0017
rs6704768	2	233592501	A	G	0.53	-0.0766	0.0109	2.06E-12	49.4	0.0029
rs76355118	2	149412005	G	A	0.04	0.1544	0.0278	2.78E-08	30.8	0.0019
rs11693528	2	200736507	G	C	0.19	0.1028	0.0136	4.73E-14	57.1	0.0032
rs1509378	2	22754466	G	A	0.65	-0.0692	0.0119	5.39E-09	33.8	0.0022
rs17194490	3	2547786	T	G	0.17	0.0966	0.0148	6.38E-11	42.6	0.0026
rs2535627	3	52845105	C	T	0.50	-0.0704	0.0109	1.17E-10	41.7	0.0025
rs6439649	3	136371691	T	G	0.56	0.0710	0.0111	1.37E-10	40.9	0.0025
rs75968099	3	36858583	T	C	0.35	0.0801	0.0114	2.31E-12	49.4	0.0029
rs832190	3	63842629	T	C	0.61	-0.0699	0.0113	5.73E-10	38.3	0.0023
rs34796896	3	180623255	A	G	0.19	-0.0822	0.0135	1.23E-09	37.1	0.0021
rs17073903	4	183632181	G	A	0.15	0.0814	0.0148	3.92E-08	30.3	0.0017
rs35225200	4	103146888	C	A	0.09	0.1448	0.0203	9.56E-13	50.9	0.0033
rs1106568	4	176861301	A	G	0.74	-0.0694	0.0125	2.85E-08	30.8	0.0018
rs215411	4	23423603	A	T	0.33	0.0692	0.0115	1.68E-09	36.2	0.0021
rs3849046	5	137851192	T	C	0.51	0.0625	0.0109	1.04E-08	32.9	0.0020
rs76091702	5	152482963	T	C	0.06	0.1293	0.0236	4.49E-08	30.0	0.0017
rs111294930	5	152177121	G	A	0.22	-0.0877	0.0143	9.29E-10	37.6	0.0026
rs11740474	5	153680747	T	A	0.41	0.0627	0.0112	2.00E-08	31.3	0.0019
rs16867576	5	88746331	G	A	0.12	-0.0958	0.0170	1.60E-08	31.8	0.0019
rs4391122	5	60598543	G	A	0.46	0.0780	0.0109	8.90E-13	51.2	0.0030
rs147439581	6	32486366	T	C	0.26	-0.0853	0.0149	1.10E-08	32.8	0.0028
rs113397282	6	32488050	C	T	0.15	-0.1450	0.0176	1.80E-16	67.9	0.0054

rs12055602	6	32622382	C	T	0.05	-0.1723	0.0220	4.69E-15	61.3	0.0030
rs117074560	6	96459651	T	C	0.04	-0.1566	0.0277	1.66E-08	32.0	0.0017
rs169738	6	33537546	G	A	0.62	0.0745	0.0112	2.67E-11	44.2	0.0026
rs1046089	6	31602967	A	G	0.36	-0.0894	0.0122	2.40E-13	53.7	0.0037
rs186545906	6	32478432	A	G	0.11	-0.1188	0.0196	1.41E-09	36.7	0.0027
rs3798869	6	84328660	A	G	0.46	-0.0668	0.0110	1.09E-09	36.9	0.0022
rs1233578	6	28712247	G	A	0.15	-0.1964	0.0164	6.17E-33	143.4	0.0098
rs3094691	6	31274693	A	G	0.46	0.0836	0.0127	4.20E-11	43.3	0.0035
rs145607970	6	32624017	A	C	0.07	-0.1679	0.0260	1.16E-10	41.7	0.0035
rs1339227	6	73155701	T	C	0.36	-0.0633	0.0114	3.06E-08	30.8	0.0018
rs114012503	6	32537489	G	A	0.14	-0.1060	0.0163	8.18E-11	42.3	0.0026
rs17529963	7	137079172	C	T	0.34	-0.0629	0.0114	3.24E-08	30.4	0.0018
rs12704290	7	86427626	A	G	0.13	-0.1061	0.0168	2.59E-10	39.9	0.0026
rs13240464	7	110898915	C	T	0.38	-0.0807	0.0116	3.12E-12	48.4	0.0031
rs7801375	7	131567263	G	A	0.85	0.0830	0.0150	2.88E-08	30.6	0.0018
rs58120505	7	2029867	C	T	0.41	-0.0822	0.0111	1.26E-13	54.8	0.0033
rs7811681	7	104928869	G	A	0.65	-0.0687	0.0114	1.63E-09	36.3	0.0022
rs36068923	8	111485761	G	A	0.19	0.0835	0.0134	4.14E-10	38.8	0.0021
rs73191547	8	10033425	T	A	0.29	0.0669	0.0115	6.13E-09	33.8	0.0018
rs10108725	8	4191202	T	C	0.22	0.0732	0.0133	3.32E-08	30.3	0.0019
rs4129585	8	143312933	C	A	0.56	-0.0793	0.0109	3.61E-13	52.9	0.0031
rs73229090	8	27442127	A	C	0.12	-0.0995	0.0177	1.95E-08	31.6	0.0021
rs13261481	8	60701801	G	T	0.59	-0.0624	0.0110	1.66E-08	32.2	0.0019
rs7819570	8	89588626	T	G	0.21	0.0765	0.0140	4.47E-08	29.9	0.0019
rs17687067	8	17036201	C	A	0.22	0.0763	0.0139	4.49E-08	30.1	0.0020
rs11139497	9	84739941	A	T	0.34	0.0656	0.0118	2.65E-08	30.9	0.0019
rs11191419	10	104612335	A	T	0.33	-0.1016	0.0118	6.69E-18	74.1	0.0046
rs79780963	10	104952499	T	C	0.09	-0.1597	0.0195	2.79E-16	67.1	0.0041
rs7893279	10	18745105	G	T	0.13	-0.1124	0.0175	1.24E-10	41.3	0.0028
rs73034295	11	133822133	A	G	0.21	-0.0910	0.0137	2.85E-11	44.1	0.0027
rs2514218	11	113392994	T	C	0.33	-0.0722	0.0116	4.64E-10	38.7	0.0023
rs10791097	11	130718630	G	T	0.52	-0.0766	0.0109	2.05E-12	49.4	0.0029
rs35324223	11	46402852	G	A	0.18	0.0920	0.0145	2.04E-10	40.3	0.0025
rs11027857	11	24403620	A	G	0.59	0.0640	0.0109	3.67E-09	34.5	0.0020
rs55661361	11	124613957	A	G	0.34	-0.0788	0.0116	1.04E-11	46.2	0.0028
rs1024582	12	2402246	G	A	0.66	-0.0989	0.0115	6.27E-18	74.0	0.0044
rs4766428	12	110723245	T	C	0.46	0.0694	0.0112	6.12E-10	38.4	0.0024
rs12826178	12	57622371	T	G	0.07	-0.1682	0.0244	5.70E-12	47.5	0.0038
rs1615350	12	123650335	T	C	0.73	-0.0851	0.0123	4.26E-12	47.9	0.0028
rs2332700	14	72417326	G	C	0.76	-0.0771	0.0125	7.38E-10	38.0	0.0022
rs12887734	14	104046834	T	G	0.27	0.0883	0.0121	3.72E-13	53.3	0.0031
rs67981189	14	71472226	G	A	0.33	0.0698	0.0118	3.75E-09	35.0	0.0021

rs1191551	14	30000405	G	T	0.78	-0.0717	0.0131	4.21E-08	30.0	0.0017
rs2693698	14	99719219	G	A	0.55	0.0617	0.0111	2.99E-08	30.9	0.0019
rs950169	15	84706461	T	C	0.28	-0.0787	0.0123	1.39E-10	40.9	0.0025
rs4702	15	91426560	A	G	0.57	-0.0805	0.0115	2.62E-12	49.0	0.0032
rs28681284	15	78908565	T	C	0.21	-0.1016	0.0141	6.35E-13	51.9	0.0035
rs783540	15	83254708	G	A	0.43	0.0599	0.0110	4.77E-08	29.7	0.0018
rs2414718	15	61863133	A	G	0.62	0.0698	0.0110	1.98E-10	40.3	0.0023
rs9922678	16	9946319	A	G	0.31	0.0684	0.0118	6.18E-09	33.6	0.0020
rs8055219	16	13753384	A	G	0.26	0.0770	0.0127	1.45E-09	36.8	0.0023
rs12691307	16	29939877	G	A	0.48	-0.0719	0.0113	2.03E-10	40.5	0.0026
rs12932476	16	63709630	G	C	0.51	-0.0597	0.0109	4.62E-08	30.0	0.0018
rs4523957	17	2208899	T	G	0.60	0.0697	0.0115	1.40E-09	36.7	0.0023
rs11658257	17	17956459	C	G	0.57	-0.0662	0.0115	8.34E-09	33.1	0.0021
rs9966779	18	53620456	T	C	0.05	-0.1329	0.0231	8.56E-09	33.1	0.0017
rs11874716	18	52750688	G	T	0.39	-0.0672	0.0110	1.01E-09	37.3	0.0021
rs9636107	18	53200117	G	A	0.50	0.0796	0.0108	2.17E-13	54.3	0.0032
rs715170	18	53795514	T	C	0.29	-0.0669	0.0122	4.65E-08	30.1	0.0019
rs2053079	19	30987423	G	A	0.27	0.0718	0.0127	1.74E-08	32.0	0.0020
rs72986630	19	11849736	T	C	0.05	0.1459	0.0266	4.12E-08	30.1	0.0022
rs2315283	19	19480575	C	T	0.66	-0.0664	0.0115	6.64E-09	33.3	0.0020
rs2103655	20	37425958	A	G	0.66	0.0766	0.0119	1.24E-10	41.4	0.0026
rs133047	22	41027819	C	T	0.89	-0.1096	0.0182	1.70E-09	36.3	0.0023
rs760648	22	42571028	A	G	0.42	0.0758	0.0118	1.27E-10	41.3	0.0028
rs5995756	22	40000313	C	T	0.54	-0.0725	0.0109	2.91E-11	44.2	0.0026

Table 10. Proxies used for SNPs that was not available for an outcome.

Sleep traits	SNP	Proxies	Correlated Alleles	R^2
Morning diurnal preference	rs2072727 (T/C)	rs2425670	T=G, C=A	1.0
	rs80097534 (G/T)	rs77371258	G=C, T=A	1.0
	rs12249410*	None	—	—
	rs497338 (C/T)	rs1769216	C=A, T=T	0.9605
	rs4535583 (C/T)	rs2213552	C=T, T=C	0.7632
	rs35588117 (A/G)	rs17681153	A=T, G=C	0.6682
Sleep duration	rs1939455 (G/T)	rs35450663	G=C, T=T	0.6592
	rs2072727 (T/C)	rs2425670	T=G, C=A	1.0
	rs7115226 (C/A)	rs9736857	C=C, A=T	0.6111
Short sleep duration	rs12963463 (C/T)	rs12969536	C=C, T=G	0.7964
	rs7939345 (T/G)	rs1997308	T=A, G=G	0.6590
Daytime napping	rs35011311 (G/T)	rs1596437	G=C, T=T	0.7772
Insomnia	rs4577309 (A/G)	rs2043989	A=C, G=G	0.6846
Schizophrenia	rs147439581*	None	—	—
	rs113397282*	None	—	—
	rs12055602*	None	—	—
	rs186545906*	None	—	—
	rs11685299 (C/A)	rs11685253	C=C, A=T	1.0
	rs145607970 (C/A)	rs9274063	C=G, A=A	1.0
	rs1339227 (C/T)	rs2789605	C=T, T=C	1.0
	rs13261481 (T/G)	rs10808700	T=C, G=G	1.0
	rs7819570 (G/T)	rs7833613	G=A, T=T	1.0
	rs11658257 (G/C)	rs6502634	G=T, C=C	1.0
	rs783540 (A/G)	rs783532	A=C, G=T	1.0
	rs114012503[#]	None	—	—

* There were no proxies with R^2>0.6 or no proxies were available for outcome.

[#] The SNPs was not available proxy when outcome was daytime napping.

Table 11. Mendelian randomization association of causal effects of sleep traits on schizophrenia.

**Sleep traits**	**SNP**	**Method**	**OR**	**95% CI**	**P-value**
Morning diurnal preference	315	IVW (multiplicative random effects)	0.839	(0.724, 0.973)	0.020
		Simple median	0.878	(0.765, 1.007)	0.062
		Weighted median	0.866	(0.747, 1.004)	0.060
Sleep duration	74	IVW (multiplicative random effects)	1.562	(1.056, 2.310)	0.026
		Simple median	1.718	(1.266, 2.331)	0.001
		Weighted median	1.547	(1.107, 2.162)	0.011
Short sleep duration	26	IVW (multiplicative random effects)	1.046	(0.725, 1.509)	0.809
		Simple median	0.829	(0.639, 1.076)	0.158
		Weighted median	0.766	(0.593, 0.988)	0.040
Long sleep duration	7	IVW (multiplicative random effects)	1.404	(1.137, 1.733)	0.002
		Simple median	1.272	(0.988, 1.638)	0.062
		Weighted median	1.262	(0.995, 1.601)	0.055
Daytime napping	105	IVW (multiplicative random effects)	2.051	(1.283, 3.279)	0.003
		Simple median	2.047	(1.315, 3.186)	0.001
		Weighted median	2.151	(1.356, 3.412)	0.001
Daytime sleepiness	40	IVW (multiplicative random effects)	1.766	(0.472, 6.615)	0.399
		Simple median	1.852	(0.762, 4.500)	0.174
		Weighted median	2.382	(0.996, 5.695)	0.051
Insomnia	52	IVW (multiplicative random effects)	0.776	(0.384, 1.569)	0.481
		Simple median	0.575	(0.317, 1.043)	0.069
		Weighted median	0.465	(0.251, 0.863)	0.015

Table 12. Heterogeneity and MR-Egger test for Horizontal pleiotropy.

**Sleep traits**	**Heterogeneity**			
	**Q**	**O_df**	**P**	**I² (%)**
Morning diurnal preference	1132	314	9.13E-93	72.3
Sleep duration	364	73	2.73E-40	79.9
Short sleep duration	166	25	7.88E-23	84.9
Long sleep duration	9.8	6	0.134	38.8
Daytime napping	352	104	9.69E-29	70.5
Daytime sleepiness	256	39	1.05E-33	84.8
Insomnia	256	39	1.05E-33	84.8
	**MR-Egger test for directional pleiotropy**			
	**Egger_intercept**	**Standard error**	**P**	
Morning diurnal preference	-0.001	0.004	0.886	
Sleep duration	0.002	0.013	0.857	
Short sleep duration	-0.017	0.026	0.526	
Long sleep duration	0.004	0.022	0.862	
Daytime napping	-0.003	0.008	0.697	
Daytime sleepiness	-0.011	0.022	0.622	
Insomnia	-0.011	0.022	0.622	

Table 13. Mendelian randomization association of causal effects of sleep traits on schizophrenia after correction for outliers.

**Sleep traits**	**SNP**	**Method**	**OR**	**95% CI**	**P-value**
Morning diurnal preference	288	IVW (multiplicative random effects)	0.840	(0.720, 0.979)	0.026
		IVW (fixed effects)	0.840	(0.775, 0.910)	2.14E-05
		Simple median	0.869	(0.748, 1.010)	0.067
		Weighted median	0.876	(0.755, 1.016)	0.080
Sleep duration	64	IVW (multiplicative random effects)	1.761	(1.181, 2.625)	0.005
		IVW (fixed effects)	1.761	(1.460, 2.124)	3.35E-09
		Simple median	1.912	(1.359, 2.691)	2.00E-04
		Weighted median	1.550	(1.075, 2.237)	0.019
Short sleep duration	20	IVW (multiplicative random effects)	1.054	(0.776, 1.433)	0.736
		IVW (fixed effects)	1.054	(0.896, 1.240)	0.525
		Simple median	0.928	(0.706, 1.218)	0.589
		Weighted median	0.776	(0.596, 1.010)	0.059
Daytime napping	99	IVW (multiplicative random effects)	2.130	(1.307, 3.470)	0.002
		IVW (fixed effects)	2.130	(1.640, 2.766)	1.46E-08
		Simple median	2.124	(1.340, 3.365)	0.001
		Weighted median	2.256	(1.395, 3.646)	0.001
Daytime sleepiness	30	IVW (multiplicative random effects)	2.821	(0.578, 13.774)	0.200
		IVW (fixed effects)	2.821	(1.555, 5.115)	6.39E-04
		Simple median	2.384	(0.920, 6.176)	0.074
		Weighted median	2.405	(0.895, 6.467)	0.082
Insomnia	46	IVW (multiplicative random effects)	1.158	(0.573, 2.341)	0.683
		IVW (fixed effects)	1.158	(0.794, 1.690)	0.446
		Simple median	0.892	(0.475, 1.675)	0.722
		Weighted median	0.865	(0.448, 1.670)	0.665

Table 14. Mendelian randomization association of causal effects of schizophrenia on sleep traits

Exposure	Sleep traits	SNP	Method	OR	95% CI	P-value
	Morning diurnal preference	101	IVW (multiplicative random effects)	0.984	(0.969, 1.000)	0.048
			Simple median	0.980	(0.966, 0.994)	0.005
			Weighted median	0.981	(0.967, 0.994)	0.005
	Sleep duration	101	IVW (multiplicative random effects)	1.026	(1.014, 1.038)	1.26E-05
			Simple median	1.026	(1.015, 1.037)	1.60E-06
			Weighted median	1.028	(1.018, 1.039)	1.09E-07
	Short sleep duration	101	IVW (multiplicative random effects)	0.994	(0.989, 0.998)	0.006
			Simple median	0.995	(0.991, 1.000)	0.043
			Weighted median	0.993	(0.989, 0.997)	0.001
	Long sleep duration	101	IVW (multiplicative random effects)	1.005	(1.002, 1.008)	2.72E-04
schizophrenia			Simple median	1.006	(1.003, 1.009)	8.06E-05
			Weighted median	1.007	(1.003, 1.010)	3.65E-05
	Daytime napping	100	IVW (multiplicative random effects)	1.010	(1.003, 1.017)	0.006
			Simple median	1.011	(1.005, 1.017)	4.89E-04
			Weighted median	1.012	(1.006, 1.018)	1.93E-04
	Daytime sleepiness	101	IVW (multiplicative random effects)	1.001	(0.996, 1.006)	0.751
			Simple median	0.999	(0.994, 1.004)	0.711
			Weighted median	1.000	(0.995, 1.005)	0.979
	Insomnia	101	IVW (multiplicative random effects)	1.002	(0.995, 1.008)	0.618
			Simple median	1.003	(0.996, 1.010)	0.401
			Weighted median	1.003	(0.996, 1.010)	0.415

Table 15. Heterogeneity and MR-Egger test for Horizontal pleiotropy.

Exposure	Sleep traits	Heterogeneity			
		Q	O_df	P	I² (%)
schizophrenia	Morning diurnal preference	446	100	2.95E-45	
	Sleep duration	348	100	2.70E-29	
	Short sleep duration	311	100	1.50E-23	
	Long sleep duration	181	100	1.24E-06	
	Daytime napping	446	99	1.52E-45	
	Daytime sleepiness	329	100	3.40E-26	
	Insomnia	306	100	8.39E-23	
		**MR-Egger test for directional pleiotropy**			
		**Egger_intercept**	**Standard error**	**P**	
schizophrenia	Morning diurnal preference	0.003	0.002	0.151	
	Sleep duration	-0.0004	0.002	0.846	
	Short sleep duration	-0.0002	0.001	0.772	
	Long sleep duration	-0.0001	0.000	0.792	
	Daytime napping	-0.001	0.001	0.464	
	Daytime sleepiness	-0.001	0.001	0.151	
	Insomnia	-0.002	0.001	0.102	

Table 16. Mendelian randomization association of causal effects of schizophrenia on sleep traits after correction for outliers.

Exposure	Sleep traits	SNP	Method	OR	95% CI	P-value
schizophrenia	Morning diurnal preference	91	IVW (multiplicative random effects)	0.983	(0.967, 0.999)	0.042
			IVW (fixed effects)	0.983	(0.976, 0.991)	1.39E-05
			Simple median	0.980	(0.966, 0.994)	0.006
			Weighted median	0.981	(0.968, 0.996)	0.011
	Sleep duration	94	IVW (multiplicative random effects)	1.026	(1.013, 1.038)	4.03E-05
			IVW (fixed effects)	1.026	(1.019, 1.032)	9.12E-15
			Simple median	1.026	(1.015, 1.037)	4.73E-06
			Weighted median	1.028	(1.017, 1.040)	7.20E-07
	Short sleep duration	98	IVW (multiplicative random effects)	0.994	(0.989, 0.998)	0.008
			IVW (fixed effects)	0.994	(0.991, 0.996)	2.35E-06
			Simple median	0.996	(0.991, 1.000)	0.050
			Weighted median	0.993	(0.989, 0.998)	0.005
	Long sleep duration	100	IVW (multiplicative random effects)	1.005	(1.002, 1.008)	3.46E-04
			IVW (fixed effects)	1.005	(1.003, 1.007)	1.31E-06
			Simple median	1.006	(1.003, 1.009)	2.56E-05
			Weighted median	1.007	(1.003, 1.010)	5.50E-05
	Daytime napping	90	IVW (multiplicative random effects)	1.009	(1.001, 1.016)	0.022
			IVW (fixed effects)	1.009	(1.005, 1.012)	1.00E-06
			Simple median	1.005	(0.998, 1.011)	0.180
			Weighted median	1.009	(1.002, 1.016)	0.009
	Daytime sleepiness	96	IVW (multiplicative random effects)	1.001	(0.995, 1.006)	0.850
			IVW (fixed effects)	1.001	(0.998, 1.003)	0.728
			Simple median	0.998	(0.994, 1.003)	0.479
			Weighted median	1.000	(0.995, 1.004)	0.914
	Insomnia	97	IVW (multiplicative random effects)	1.001	(0.995, 1.008)	0.689
			IVW (fixed effects)	1.001	(0.997, 1.005)	0.496
			Simple median	1.003	(0.996, 1.010)	0.389
			Weighted median	1.003	(0.996, 1.009)	0.440
